# Permeability-driven pressure and cell proliferation control lumen morphogenesis in pancreatic organoids

**DOI:** 10.1038/s41556-025-01832-5

**Published:** 2025-12-19

**Authors:** Byung Ho Lee, Kana Fuji, Heike Petzold, Phil Seymour, Siham Yennek, Coline Schewin, Allison Lewis, Daniel Riveline, Tetsuya Hiraiwa, Masaki Sano, Anne Grapin-Botton

**Affiliations:** 1 Max Planck Institute of Molecular Cell Biology and Genetics, Dresden, Germany; 2 Universal Biology Institute, Graduate School of Science, The University of Tokyo, Tokyo, Japan; 3 The Novo Nordisk Foundation Center for Stem Cell Biology, Copenhagen, Denmark; 4 IGBMC, Université de Strasbourg, CNRS, INSERM, Institut de Génétique et de Biologie Moléculaire et Cellulaire, Illkirch, France; 5 Institute of Physics, Academia Sinica, Taipei, Taiwan; 6 Mechanobiology Institute, National University of, Singapore, Singapore; 7 Institute of Natural Sciences, Shanghai Jiao Tong University, Shanghai, China; 8 Cluster of Excellence Physics of Life, TU Dresden, Dresden, Germany; 9 Paul Langerhans Institute Dresden of the Helmholtz Zentrum München at the University Clinic Carl Gustav Carus of Technische Universität Dresden, Helmholtz Zentrum München, Neuherberg, Germany

**Keywords:** Morphogenesis, Cell biology

## Abstract

Lumen formation in organ epithelia involves processes such as polarization, secretion, exocytosis and contractility, but what controls lumen shape remains unclear. Here we study how lumina develop spherical or complex structures using pancreatic organoids. Combining computational phase-field modelling and experiments, we found that lumen morphology depends on the balance between cell cycle duration and lumen pressure, low pressure and high proliferation produce complex shapes. Manipulating proliferation and lumen pressure can alter or reverse lumen development both in silico and in vitro. Increasing epithelial permeability reduces lumen pressure, converting from spherical to complex lumina. During pancreas development, the epithelium is initially permeable and becomes sealed, experimentally increasing permeability at late stages impairs ductal morphogenesis. Overall, our work underscores how proliferation, pressure and permeability orchestrate lumen shape, offering insights for tissue engineering and cystic disease treatment.

## Abstract (web summary)

Using pancreatic organoids, Lee et al. show that the balance between epithelial tissue permeability-driven lumenal pressure and cell proliferation affects ductal morphogenesis.

## Main

Internal organs frequently comprise epithelia that delineate fluid-filled lumina. These lumina vary in shape, ranging from sacs such as the bladder to single tubes such as the intestine or complex networks as seen in the kidney or in many glands including the pancreas^1–3^. Studies exploring lumen formation have leveraged various model systems, including zebrafish, *Drosophila* and mouse, to understand how these luminal and ductal structures form^4^. These structures play a pivotal role in organ functionality, serving as essential transport and delivery networks; any dysmorphogenesis in these structures can lead to severe pathological conditions^5^.

Mechanistic studies of lumen morphogenesis have focused primarily on the mechanisms of polarity acquisition and lumen growth, notably using cell lines cultured in three dimensions such as the Madin–Darby canine kidney (MDCK) system^6^. Though this yielded valuable insight, lumen morphology typically presents as a single sphere in these systems. In addition, organoid models, which more closely mimic physiological organs, often feature a single spherical lumen^6–10^. However, they can exhibit more complex geometries and topologies such as outpocketings around a spherical core^11^, multiple small lumina^10,12^ or networks of thin tubes^13,14^, whose formation and diversity deserve more mechanistic understanding.

The intricate process of lumen morphogenesis involves multiple cellular mechanisms such as epithelial polarization, secretion, vesicle trafficking and fusion, and cortical contractility, as well as cell death and rearrangement^8,15^. Numerous proteins controlling these processes have been identified. In addition, recent focus on the physics of lumen formation, notably the balance of luminal forces, such as hydrostatic pressure and cell mechanics has provided a more biophysical view of lumenogenesis^9,11,12,16–23^. These studies have largely been limited to spherical lumina. Pancreatic organoids can form either large spherical lumen or narrow complex interconnected lumen structures, depending on the culture medium^13^. In this study, we investigate how morphological trajectories arise and can be altered or reversed from a fundamental mechanical perspective. Combining experimental insights with multicellular phase-field modelling, our assessment of proliferation and lumen pressure as well as targeted interventions unveil how the balance between cell cycle rate and luminal pressure orchestrates the diverse spectrum of lumen morphologies. Our work shows that the leaky epithelium found in organoids and in the early pancreas in vivo prevents high luminal pressure and together with fast proliferation conditions enables the formation of narrow complex interconnected lumen similar to those found in vivo.

## Results

### Two distinct morphological lumen trajectories in pancreas organoids

**Fig. 1:**
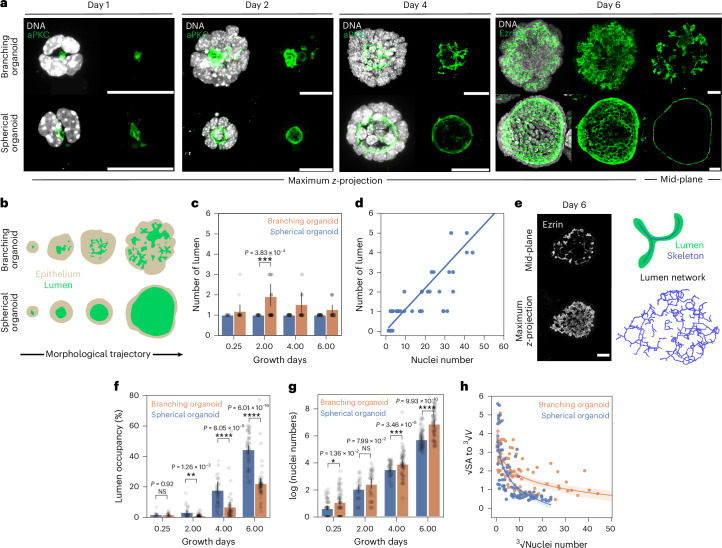
Divergent lumen morphology and topology trajectories in pancreatic branching and spherical organoids. **a**, Immunofluorescence images of branching and spherical organoids at days 1, 2, 4 and 6 of culture growth in Matrigel. The green colour is the apical marker aPKC (day 1–4) and Ezrin (day 6) and the white is DNA. Scale bar, 20 µm. **b**, A schematic representing the morphological trajectories of branching and spherical organoids. Beige, epithelium; green, lumen. **c**, The number of lumina (quantified in three dimensions) of branching and spherical organoids at various days of culture growth. The data are presented as the mean ± 95% confidence intervals from five independent experiments for growth days 0.25 and 2 and three independent experiments for growth days 4 and 6 (*n* = 154 spherical organoids; *n* = 97 branching organoids). **d**, The relationship between lumen number and cell number of branching organoids at day 2; shown with a linear regression. The data points represent individual organoids (*n* = 50 branching organoids) from three independent experiments. **e**, Left: immunofluorescence mid-plane and maximum-intensity projected images showing branching organoids at growth day 6 with Ezrin marking the lumen. Right: a schematic showing the skeletonization of a lumen (top) to obtain a 3D lumen network (bottom) depicting lumen topology. Scale bar, 20 µm. **f**, Lumen occupancy (percentage of total organoid volume; quantified in three dimensions) of branching and spherical organoids at various days of culture growth. The data are presented as the mean ± 95% confidence interval from five independent experiments for growth days 0.25 and 2 and three independent experiments for growth days 4 and 6 (*n* = 155 spherical organoids; *n* = 151 branching organoids). **g**, The number of nuclei (expressed as log[number of nuclei]) in branching and spherical organoids at various days of culture growth. The data are presented as the mean ± 95% confidence interval from five independent experiments for growth days 0.25 and 2 and three independent experiments for growth days 4 and 6 (*n* = 238 branching organoids; *n* = 198 spherical organoids). **h**, The relationship between lumen surface-to-volume ratio ($\sqrt{\mathrm{surface}\,\mathrm{area}}:\root{{3}}\of{\mathrm{volume}}$) and the number of nuclei ($\root{{3}}\of{\mathrm{nuclei}\,\mathrm{number}}$) per organoid; the linear regression ± 95% confidence interval. The data are presented from from five independent experiments for growth days 0.25 and 2 and three independent experiments for growth days 4 and 6 (*n* = 91 branching organoids; *n* = 91 spherical organoids). The *P* values were determined by a two-sided Mann–Whitney test for **c**, **f** and **g**. NS, not significant. Source data

**Extended Data Fig. 1:**
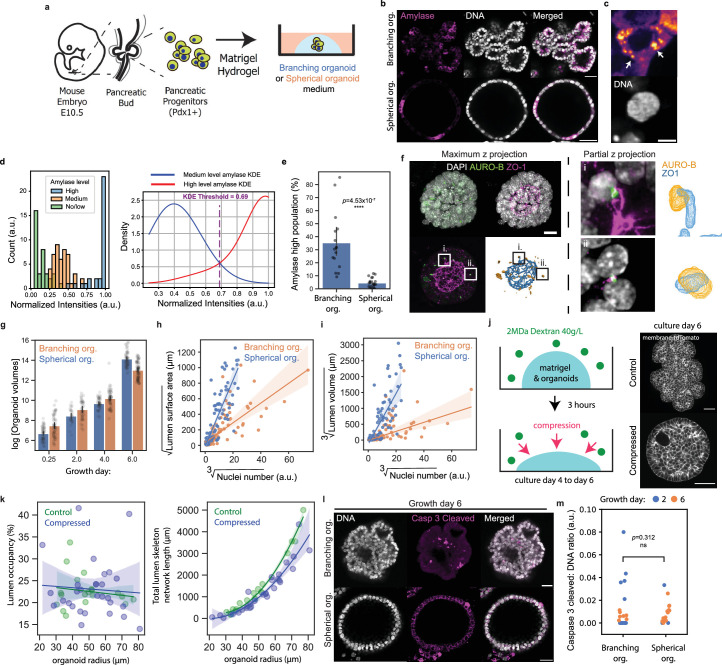
Initiation and evolution of lumen formation in pancreatic branching and spherical organoids. (**a**) Schematic of the organoid protocol: collect E10.5 embryos, dissect dorsal pancreatic buds, dissociate to cell aggregates, embed in Matrigel, and culture in organoid medium. **(b)** Immunofluorescence images of branching and spherical organoids stained for amylase and DNA (Hoechst). Scale bar = 40 µm. **(c)** High-magnification immunofluorescence image of an organoid stained for amylase and DNA (Hoechst); white arrows indicate cytoplasmic amylase puncta. Scale bar = 5 µm. **(d)** Left: histogram of normalized amylase intensities (normalized to the maximum intensity across the cell population); the legend indicates visually inspected categories (high/medium/low). Data pooled from two independent experiments (n = 252 cells). Right: combined Kernel Density Estimation (KDE) plots of high-level (red) and medium-level (blue) amylase intensities. The cut-off (vertical dashed line) between high and medium levels was obtained by KDE-based thresholding to assign high-amylase cells within an organoid (n = 33 high-amylase; n = 47 medium-amylase cells). **(e)** Percentage of cells expressing high amylase per organoid in branching and spherical organoids. Mean ± standard deviation, data pooled from two independent experiments (n = 21 spherical organoids; n = 17 branching organoids). **(f)** Left: maximum-projected immunofluorescence images of spherical organoids stained with DAPI (gray, nuclei), Aurora-B (green, abscission point), and ZO-1 (magenta, sub-apical tight junctions), with corresponding mesh representations of segmented Aurora-B and ZO-1. Right: partial z-projected zoom-in showing co-localization of Aurora-B and ZO-1. Scale bar = 10 µm. **(g)** Organoid volume (log-transformed) for branching and spherical organoids. Mean ± 95% confidence interval of data from five independent experiments for growth days 0.25 and 2, and three independent experiments for growth days 4 and 6 (n = 219 branching organoids; n = 197 spherical organoids). **(h)** Relationship between $\sqrt{{lumen\; surface\; area}}$ and $\root{{3}}\of{{nuclei\; number}}$ for branching and spherical organoids. Linear regression ± 95% confidence interval; data points represent individual organoids, pooled from five independent experiments for growth days 0.25 and 2, and three independent experiments for growth days 4 and 6 (n = 91 branching; n = 91 spherical). **(i)** Relationship between $\root{{3}}\of{{lumen\; volume}}$ and $\root{{3}}\of{{nuclei\; number}}$ for branching and spherical organoids. Linear regression ± 95% confidence interval; data points represent individual organoids, pooled from five independent experiments for growth days 0.25 and 2, and three independent experiments for growth days 4 and 6 (n = 91 branching; n = 91 spherical). **(j)** Left: schematic of the compression assay for branching organoids. Right: immunofluorescence images of membrane-tdTomato expressing branching organoids under control (non-compressed) and compressed conditions at day 6. **(k)** 3D lumen occupancy (left) and total lumen skeleton length (right) in branching organoids under non-compressed and compressed conditions. Linear regression (left) and polynomial fit (order 2; right) ± 95% confidence interval; data pooled from two independent experiments (n = 18 non-compressed; n = 29 compressed). **(l)** Immunofluorescence images of branching and spherical organoids stained for cleaved caspase-3 and DNA (Hoechst). Scale bar = 30 µm. **(m)** A quantification of cleaved caspase-3–positive:DNA ratio per organoid. Data pooled from two independent experiments (n = 24 branching organoids; n = 18 spherical organoids). *P* values were determined by two-sided Mann-Whitney test (**e, m**). Source data

Our previous work has shown that dissociated cells from embryonic day 10.5 (E10.5) pancreatic buds can generate spherical single-lumen and interconnected complex-lumen organoids in different media (hereafter, called spherical organoids and branching organoids for simplicity)^13^ (Extended Data Fig. 1a). Specifically, the epithelium of the branching organoids forms a multilayered and branched structure by day 6, whereas the spherical organoids maintain their characteristic epithelium monolayer throughout culture growth (Fig. 1a). Both progenitors, acinar cells and endocrine cells were found in these organoids though in different proportions^13^ (Extended Data Fig. 1b–e).

Divergence in the lumen morphology became evident from day 2 onwards, where spherical organoids typically developed and maintained a single lumen throughout culture growth, whereas branching organoids frequently formed multiple lumina (Fig. 1b,c). When multiple lumina were present, a larger star-shaped lumen and smaller peripheral lumina were observed. Our previous work has shown that lumina form in two ways in this system, either by cavitation between the small number of cells that seed a lumen or at the time of cell division at the abscission point^10^ (Extended Data Fig. 1f). On day 2, the number of lumina formed was proportional to the number of cells in a branching organoid (Fig. 1d). Notably, three-dimensional (3D) analyses showed that the average number of lumina in the branching organoids peaked on day 2 before decreasing (Fig. 1c). This revealed the progressive formation of a hyperconnected network, most probably due to the emergence of connections between lumina (Fig. 1e).

To further elucidate lumen volume evolution, we analysed lumen occupancy, which is defined as the 3D lumen volume relative to the total organoid volume (Fig. 1f). Both organoid systems showed increasing lumen occupancy over time. Yet, from day 2 onward, branching organoids consistently exhibited lower lumen occupancy compared with spherical organoids. Meanwhile, the epithelium of the branching organoids displayed higher number of cells throughout culture (Fig. 1g and Extended Data Fig. 1g, organoid volumes). Initially, the surface-to-volume ratio of lumina decreased similarly in both systems, consistent with a phase of lumen growth (Fig. 1h and Extended Data Fig. 1h,i). As the cell number increased, the branching organoids displayed higher surface-to-volume ratio than the spherical organoids, as expected for a system with multiple small lumina or more convoluted lumina (Fig. 1h). By contrast, spherical organoids minimized their surface-to-volume ratio, maintaining their spherical lumen geometry. To exclude the possibility that complex lumen morphologies arise as a secondary consequence of epithelial branching, we subjected branching organoids to osmotic compression with 2 MDa dextran at culture day 4, when branching first becomes apparent^24^ (Fig. 1a and Extended Data Fig. 1j). Despite reduced outer branching, compressed organoids maintained lumen occupancy and internal lumen-network lengths comparable to those of non-compressed branching organoids (Extended Data Fig. 1j,k), demonstrating that lumenogenesis is an intrinsic epithelial process rather than a consequence of surface branching. The investigations established here provide a foundation to address what causes differences in both topological (number of lumina) and other geometric features (lumen occupancy, surface-to-volume ratios) between spherical and branching organoids.

### Phase-field modelling and experiments reveal a key role of lumen pressure and cell cycle duration on lumina morphology

Since the increase in cell number was faster in branching organoids, we hypothesized that the creation of a new lumen at cell division might drive differences in lumen shape and topology, notably leading to an increase in the number of lumina and apical surfaces. Additionally, given studies suggesting that lumen growth can be regulated by the pressure difference between the lumen and the exterior environment, we explored whether the balance between lumen pressure and epithelial proliferation rate is a key factor distinguishing these systems^16,20,25–29^.

To connect single cell dynamics (cell growth and division) with luminal nucleation and growth, we turned to a theoretical investigation by applying phase-field multicellular modelling in two dimensions. To mimic organoids, we incorporated a force balance for each cell, considering cortical surface tension, adhesion with neighbouring cells and the neighbouring lumen with its osmotic pressure difference to the external environment, *ξ*.

**Fig. 2:**
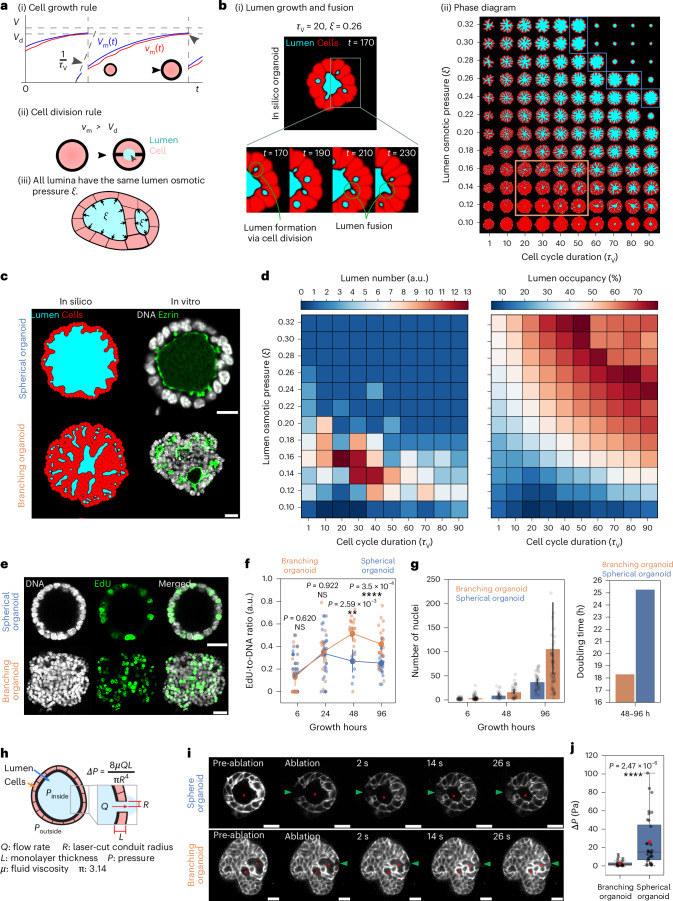
Organoids with complex lumina form at low lumen pressure difference and fast proliferation rates. **a**, Schematics describing the rules and parameters (*τ*_V_ and *ξ*) of the phase-field model governing cell growth (i), cell division (ii) and lumen osmotic pressure (iii). Each cell m divides when its actual volume *v*_m_(*t*) reaches the division volume *V*_d_. The target volume *V*_m_(*t*) is defined as *V*_m_(*t*) = target,m(*t*) in equation (1) (Methods). **b**, In silico simulation of lumen formation (via cell division), growth and fusion via cell division (i) and phase diagram capturing the spectrum of lumen and organoid morphologies as a function of *ξ* and *τ*_V_ (ii). Orange outlines mark branching organoid-like morphologies, while blue outlines denote spherical organoid-like morphologies (ii). **c**, The qualitative morphological resemblance between in vitro spherical and branching organoids and in silico organoids. Scale bar, 20 µm. **d**, Phase diagrams showing lumen number (left) and lumen occupancy (right) of in silico organoids as a function of *ξ* and *τ*_V_. **e**, Immunofluorescence images of spherical and branching organoids after 2 h of EdU labelling. Scale bar, 30 µm. **f**, A 3D quantification of the EdU:DNA ratio of spherical and branching organoids at various hours of culture growth. The data are pooled from five independent experiments for growth hours 6 and 24 and two independent experiments for growth hours 48 and 96 (*n* = 72 branching organoids; *n* = 108 spherical organoids) are presented as the mean ± 95% confidence interval. **g**, Left: a quantification of nuclei per organoid at various hours of culture growth. The data are presented as the mean ± 95% confidence interval, pooled from three independent experiments (*n* = 180 branching organoids; *n* = 129 spherical organoids). Right: a quantification of doubling time for branching and spherical organoids from 48 to 96 h of culture growth. **h**, A schematic of the laser-ablation setup and measurements used in the Hagen–Poiseuille equation to infer lumen hydrostatic pressure differences. **i**, A montage of laser-ablation experiments on branching and spherical organoids expressing membrane-tdTomato. The red asterisk is the targeted lumina (connected lumina in three dimensions for branching organoids), and the green arrowhead is the conduit created by laser ablation. Scale bar, 20 µm. **j**, The inference of lumen hydrostatic pressure (∆*P*) of branching and spherical organoid lumina on day 6 of culture. The data are represented as boxplots showing the median (centre line), the 25th and 75th percentiles (box edges) and whiskers extending to 1.5× the interquartile range. The red dots indicate the mean values (branching organoids, 2.9 Pa; spherical organoids, 25.4 Pa). The data are pooled from three independent experiments (*n* = 26 branching organoids; *n* = 32 spherical organoids). The *P* values were determined by a two-sided Mann–Whitney test for **f** and **j**. NS, not significant. Source data

As we observed differential growth in branching and spherical organoids, we included the parameter *τ*_V_, which captures the time interval between cell divisions in the absence of mechanical constraints. Moreover, the axis of cell division was determined by the force balance regulating spindle positioning in the dividing cell (see Supplementary Note and refs. ^25,30^ for more details). In brief, the cells grow in volume (*V*_*i*_) toward a target volume (*V*_target*,i*_) which is time-dependent (*t*) and can be tuned by *τ*_V_ (Fig. 2a(i)). Moreover, cell division occurs when the division volume (*V*_d_) is reached (Fig. 2a(ii)). Therefore, the *τ*_V_ parameter provides control of the cell cycle duration in silico. Meanwhile, the tuneable parameter to control lumen growth is its osmotic pressure difference to the external environment *ξ* (hereon called lumen osmotic pressure for simplicity), which is kept constant through numerical simulations and identical in all lumina for each organoid in silico (Fig. 2a(iii) and see Supplementary Note and ref. ^25^ for more details). With these rules of cell growth and division and lumen osmotic pressure, the in silico organoids grow and lumen growth & fusion can be observed (Fig. 2b(i), Supplementary Video 1 and Section 1 of Supplementary Note). The phase diagram given by the numerical simulations based on this model revealed that both *τ*_V_ and *ξ* affected lumen size, shapes and numbers (Fig. 2b(ii)).

**Extended Data Fig. 2:**
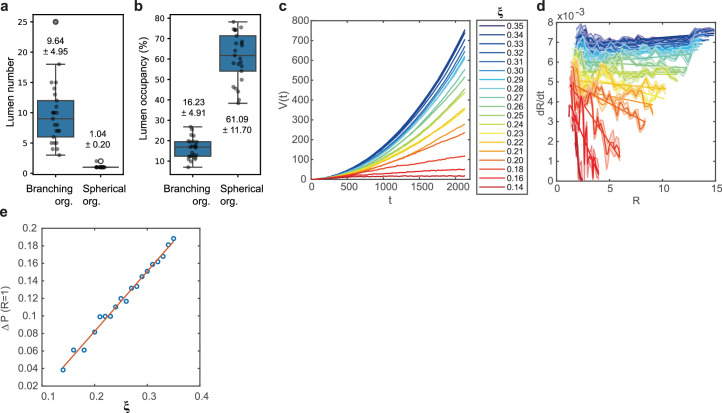
Comparison of lumen number and lumen occupancy in branching versus spherical organoids, and the *in silico∆P–ξ* relationship. **(a)** 2D lumen number quantification in branching and spherical organoids. Boxplot (median (center line), 25th and 75th percentiles (box edges), and whiskers extending to 1.5× the interquartile range) of data pooled from three independent experiments (n = 25 branching organoids; n = 25 spherical organoids). **(b)** 2D lumen occupancy quantification in branching and spherical organoids. Boxplot (median, 25th and 75th percentiles, and whiskers extending to 1.5× the interquartile range) of data pooled from three independent experiments (n = 25 branching organoids; n = 25 spherical organoids). **(c)** Time evolution of lumen volume *V(t)* from 2D simulations of cysts with varying *ξ* values (shown in the legend). Simulation parameters are the same as in Fig. 2b (*τV* = 50), with one run per ξ value. **(d)** Relationship between lumen radius growth rate (*dR/dt*) and lumen radius (R) for different osmotic pressures (*ξ*; values shown in the legend of Extended Data Fig. 2c). The lumen radius *R(t)* was calculated assuming circular (spherical in 3D) geometry as *R(t)* = *p V (t)/π*. The growth rate *dR/dt* was obtained by finite differences in time and averaged using a moving window of 30 time points. Shaded regions denote standard deviation across these data points. Straight lines represent linear fits according to Supplementary Note Equation (22). **(e)** Relationship between *ξ* and *∆P*. Estimated *∆P* as a function of *ξ* from 2D simulations of cysts using the relation between *dR/dt*, *ξ*, and *∆P* described in Supplementary Note Section 2, where values of *dR(t)/dt* were evaluated at *R* = 1 in Extended Data Fig. 2d. Red line shows the linear fit (y = −0.055 + 0.685x, R = 0.991). Source data

Multiple regions of the phase diagram recapitulated the lumen features of our in vitro organoids, specifically, high lumen number with low lumen occupancy at low *ξ* and faster *τ*_V_ and the reverse lumen phenotype at higher *ξ* and slow *τ*_V_ (branching organoids-like: orange outline; spherical organoids-like: blue outline in Fig. 2b(ii)–d and Extended Fig. 2a,b).

To experimentally test these observations and validate the model, we sought to characterize the difference in lumen hydrostatic pressure relative to the external environment (∆*P*; hereon called lumen hydrostatic pressure for simplicity) and cell cycle duration of both organoid systems. To address whether the branching organoids proliferate faster, we performed an 5-ethynyl 2′-deoxyuridine (EdU) incorporation assay, labelling cells in the S-phase within the organoids. We treated the organoids with EdU for 2 h before fixation at 6, 24, 48 and 96 hours of culture growth (Fig. 2e). To analyse the difference in proliferation, we quantified the ratio between cells that were EdU positive (S-phase) and the total number of nuclei. We observed that at 6 and 24 h, both branching and spherical organoids displayed comparable EdU:DNA ratios. However, after 24 h, the spherical organoids exhibited a consistently lower relative number of EdU positive cells compared with the branching organoids (Fig. 2f). These experiments showed that both organoid systems increase their potential to proliferate and start diverging from day 2 onward, coinciding with the similar lumen morphologies the two organoids share at day 2 (Fig. 1a,b). Moreover, no clear differences were observed in cleaved caspase 3 levels between the two organoid systems, indicating little impact of cell death (Extended Data Fig. 1l,m). Since the interval between cell divisions *τ*_V_ was found to be an important control parameter in the numerical simulations, we calculated the doubling time for both organoid systems by utilizing the average cell number in organoids at later culture days (Fig. 2g), that is, when the average morphologies of the branching and spherical organoids were different (day 2–4) (Fig. 1a,b). Assuming exponential growth and neglecting contributions from cell death, we found that the doubling time of the branching organoids was 1.4× faster than that of the spherical organoids from 48 to 96 h (Fig. 2g).

**Extended Data Fig. 3:**
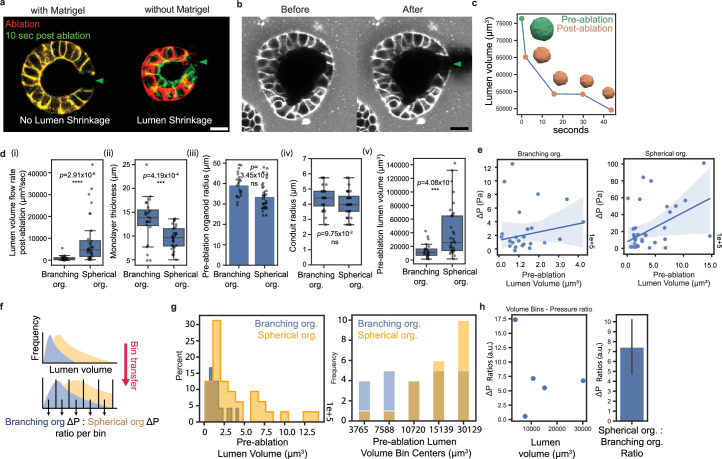
Lumen hydrostatic pressure inference of branching and spherical organoids. (**a)** Live images of laser-ablated spherical organoids expressing membrane-tdTomato in Matrigel showing no lumen shrinkage (left) and removed from Matrigel showing lumen shrinkage (right). Green arrowheads indicate conduits created by laser ablation. **(b)** Live images of CellMask-treated spherical organoids before and after laser ablation. Green arrowheads indicate conduits created by laser ablation. **(c)** 3D quantification of lumen volume over time during laser ablation experiments. Inset shows 3D meshes of segmented lumina before (green) and after (beige) ablation, illustrating lumen shrinkage. **(d)** Quantification of parameters and variables for the Hagen–Poiseuille equation for lumen hydrostatic pressure inference of branching and spherical organoids: (i) 3D lumen volume flow rate, (ii) monolayer thickness, (iii) pre-ablation organoid radius, (iv) conduit radius, and (v) pre-ablation lumen volume. Boxplots showing the median, 25th and 75th percentiles, and whiskers extending to 1.5× the interquartile range. Bar plots showing mean ± standard deviation. Data pooled from three independent experiments (n = 26 branching organoids; n = 33 spherical organoids). **(e)** Relationship between lumen hydrostatic pressure (∆P) and pre-ablation lumen volume in branching (left) and spherical (right) organoids. Linear fit ± 95% confidence interval; data pooled from three independent experiments (n = 26 branching organoids; n = 33 spherical organoids). **(f)** Schematic representation showing the calculation of relative lumen hydrostatic pressure ratios with respect to lumen volume. **(g)** Distribution of pre-ablation lumen volumes in branching (blue) and spherical (yellow) organoids (left), and corresponding bin transfers between the two populations (right). Data pooled from three independent experiments (26 branching organoids and 33 spherical organoids). **(h)** Left: Mean lumen hydrostatic pressure ratios between branching and spherical organoids per bin. Right: Average of mean lumen hydrostatic pressure ratios between branching and spherical organoids. Mean ± standard error of the mean of data pooled from five mean lumen hydrostatic pressure ratios (21 branching; 14 spherical organoids). *P* values were determined by two-sided Mann-Whitney test (**d i - v**). Source data

**Extended Data Fig. 4:**
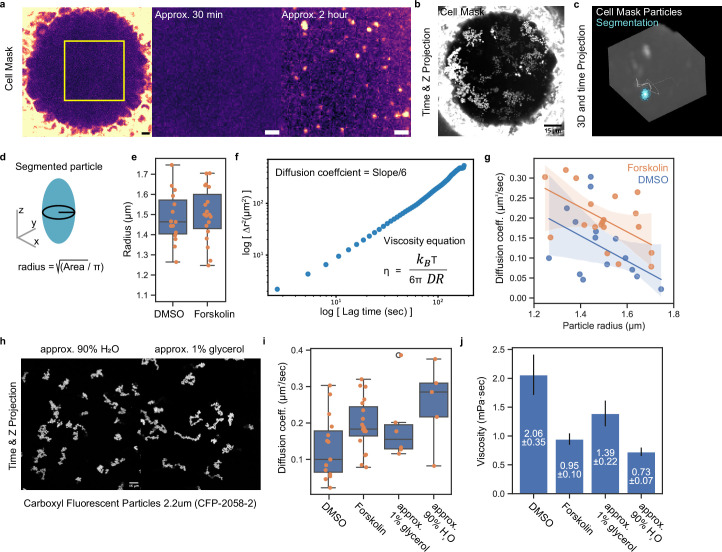
Estimation of lumen viscosity for hydrostatic pressure inference. (**a**) Live images of spherical organoids at approximately 30 minutes and 2 hours after CellMask treatment. Scale bar = 5 µm. **(b)** Time- and z-projected image of a CellMask-treated spherical organoid. Scale bar = 15 µm. **(c)** 3D projection and trajectory of a segmented CellMask-positive particle. **(d)** Schematic representing radius measurement from the 3D segmented particle. **(e)** Radius of CellMask-positive particles in DMSO- and forskolin-treated spherical organoids. Boxplot showing the median (center line), 25th and 75th percentiles (box edges), and whiskers extending to 1.5× the interquartile range. Data pooled from two independent experiments (15 particles from four DMSO-treated and 19 particles from seven forskolin-treated spherical organoids). **(f)** Example of a 3D mean squared displacement (MSD) curve of a tracked CellMask-positive particle. Inset shows equations for the diffusion coefficient and fluid viscosity, where *D* is the diffusion coefficient, *kB* is the Boltzmann constant, *T* is the absolute temperature, *η* is the dynamic viscosity, and *r* is the hydrodynamic radius of the diffusing particle. **(g)** Diffusion coefficients of particles of various sizes (radius) tracked in DMSO- and forskolin-treated organoid lumina. Linear fit ± 95% confidence interval; data pooled from two independent experiments (15 particles from four DMSO-treated and 19 particles from seven forskolin-treated spherical organoids). **(h)** Time- and z-projected images of carboxyl fluorescent particles (2.2 µm diameter) in ∼90% H_2_O and ∼1% glycerol. Scale bar = 15 µm. **(i)** Diffusion coefficients of particles tracked in DMSO- and forskolin-treated lumina and in $\sim$90% H_2_O and ∼1% glycerol. Boxplot (median (center line), 25th and 75th percentiles (box edges), and whiskers extending to 1.5× the interquartile range) of data pooled from two independent experiments (15 particles from four DMSO-treated, 19 particles from seven forskolin-treated spherical organoids, 6 ∼1% glycerol, and 5 ∼90% H_2_O samples). **(j)** Fluid viscosity of lumen samples from DMSO- and forskolin-treated spherical organoids, and from ∼90% H_2_O and ∼1% glycerol solutions. Mean ± standard error of the mean. Data pooled from two independent experiments (15 particles from four DMSO-treated, 19 particles from seven forskolin-treated spherical organoids, 6 ∼1% glycerol, and 5 ∼90% H_2_O samples). Source data

Another control parameter which played a key role in the phase diagram was *ξ*. We asked whether the branching and spherical organoids exhibited distinct lumen osmotic pressure differences. Through mathematical modelling using the phase-field model, we found that the lumen osmotic pressure shares a linear relationship with hydrostatic pressures (Section 2 of Supplementary Note and Extended Data Fig. 2c–e). With this confirmation, we utilized laser ablation at culture day 6 to estimate the lumen hydrostatic pressures in the two organoid systems by applying the Hagen–Poiseuille equation^9,20,29,31^. Laser ablation creates a conduit across the epithelium layer between the lumen and the external environment (Fig. 2h,i). Upon laser ablation, we observed a decrease in the lumen volume (measured in three dimensions) and fluid expelled from the lumen, indicating a higher lumen hydrostatic pressure than around the organoids (Extended Data Fig. 3a–c). To calculate the lumen hydrostatic pressure (∆*P*), we quantified the flow rate across the conduit based on lumen volume change, the conduit radius and epithelium thickness^9^ (Fig. 2h and Extended Data Fig. 3d). To estimate lumen fluid viscosity, we segmented and tracked CellMask-positive particles in the lumen to calculate their 3D mean squared displacement. Using these curves, we derived the diffusion coefficient and then estimated the lumen’s viscosity via the Stokes–Einstein equation (Extended Data Fig. 4a–g). With this approach, we estimated that the mean lumen fluid viscosity was 2.1 ± 0.3 mPa s (Extended Data Fig. 4h–j). As the lumina in branching organoids were too narrow to make such measurements, we assumed that the lumen viscosity in both organoid systems was similar and applied the average lumen viscosity obtained from the spherical organoids to infer ∆*P* of both systems. With the lumen viscosity, flow rate and conduit dimensions created by the laser cut, we obtained mean ∆*P* of 2.9 Pa for the branching organoids and 25.4 Pa for the spherical organoids on day 6 of culture (Fig. 2j). Moreover, we observed that the ∆*P* of the lumen in spherical organoids increased with increasing lumen volume. Notably, this was not the case for the branching organoids (Extended Data Fig. 3e). We thus compared lumen of the same size for branching and spherical organoids after binning lumen volumes (Extended Data Fig. 3f,g). This analysis revealed that ∆*P* in size-matched lumina of spherical organoids is approximately seven times higher than in branching organoids (Fig. 2j and Extended Data Fig. 3h).

To map in vitro organoids onto the in silico *ξ*–*τ*_V_ phase diagram, we quantified cross-sectional lumen number and occupancy (Extended Data Fig. 2a,b): branching organoids averaged a lumen number of 9.6 ± 4.9 and 16.2% ± 4.9% lumen occupancy, while spherical organoids averaged a lumen number of 1.0 ± 0.2 and 61.1% ± 11.7% lumen occupancy. In the phase diagram (Fig. 2b(ii),d), these qualitative and quantitative approximations place spherical organoid in the *y* axis, *ξ* = 0.3 ± 0.2 at around *τ*_V_ = 60 and branching organoids around *ξ* = 0.14 ± 0.2 range at approximately *τ*_V_ = 40. From these comparisons ratio of *ξ* is thus 2.1 in silico. As there is a linear relationship between ∆*P* and *ξ* (Section 2 of Supplementary Note and Extended Data Fig. 2c–e), we infer that spherical organoids have a 2.9× higher lumen hydrostatic pressure ∆*P* than branching organoids. Compared with the lumen hydrostatic pressure ratio observed in vitro (7.5 ± 2.7; Extended Data Fig. 3h), this ratio in the simulation is slightly smaller (within an order of magnitude), but the trend is consistent, and it is considered to be within the acceptable range of error for theoretical predictions.

Overall, these results indicate that organoids with faster proliferation and lower lumen pressure align with phase diagram regions featuring more complex lumen geometries and multilumen topologies, whereas the opposing organoid features result in a lumen that minimizes its surface-to-volume ratio to obtain a spherical morphology. This agreement between simulation and experiment strongly supports our hypothesis that the interplay between lumen pressure and cell cycle dynamics is the key determinant of lumen morphology.

### Branching organoids relax to spherical organoids upon proliferation arrest

**Fig. 3:**
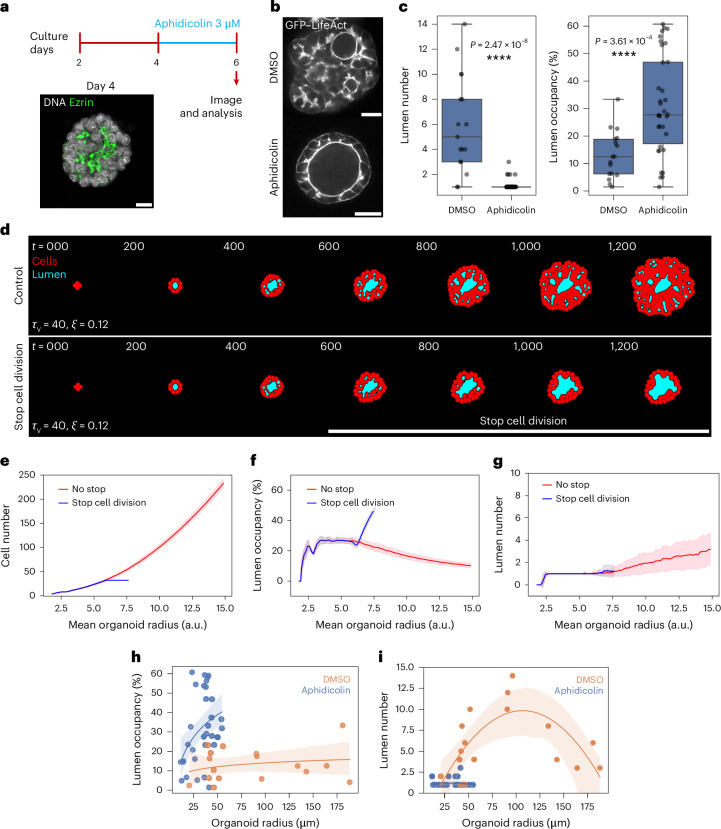
Cell cycle interference reduces the complexity of lumina. **a**, A schematic showing the experimental design of the aphidicolin assay. Scale bar, 10 µm. **b**, Immunofluorescence images of DMSO- and aphidicolin-treated branching organoids expressing GFP–LifeAct at day 6. Scale bar, 20 µm. **c**, A 2D quantification (at mid-plane) of lumen number (left) and lumen occupancy (right) per organoid of DMSO- and aphidicolin-treated branching organoids at day 6. The data are represented as boxplots showing the median (centre line), the 25th and 75th percentiles (box edges) and whiskers extending to 1.5× the interquartile range. The data are pooled from three independent experiments (*n* = 17 DMSO; *n* = 34 aphidicolin). **d**, In silico simulations of organoids under *τ*_V_ = 40 and *ξ* = 0.12. Top: simulation run without stopping cell division. Bottom: a run in which cell division was stopped at 32 cells (red, cells; blue, lumen). **e**, A quantification of the relationship between the number of cells and mean organoid radius during the simulation (red, control with no stop in cell division; blue, cell division stopped at 32 cells). *N* = 50 simulations; the bold line represents the moving average ± standard deviation error bars. **f**, A quantification of lumen occupancy versus mean organoid radius during the simulation (red, control with no stop in cell division; blue, cell division stopped at 32 cells). *N* = 50 simulations; the bold line represents the moving average ± standard deviation error bars. **g**, A quantification of lumen number versus mean organoid radius during the simulation (red, control with no stop in cell division; blue, cell division stopped at 32 cells). *N* = 50 simulations; the bold line represents the moving average ± standard deviation error bars. **h**, The relationship between lumen occupancy (in two dimensions) and organoid radius of branching organoids treated with DMSO or aphidicolin (linear regression ± 95% confidence interval). The data are pooled from three independent experiments (*n* = 17 DMSO; *n* = 34 aphidicolin). **i**, The relationship between lumen number (in two dimensions) and organoid radius of branching organoids under DMSO and aphidicolin treatment (polynomial regression (order of 2) ± 95% confidence interval). The data are pooled from three independent experiments (*n* = 17 DMSO; *n* = 34 aphidicolin). The *P* values were determined by a two-sided Mann–Whitney test for **c**. Source data

**Extended Data Fig. 5:**
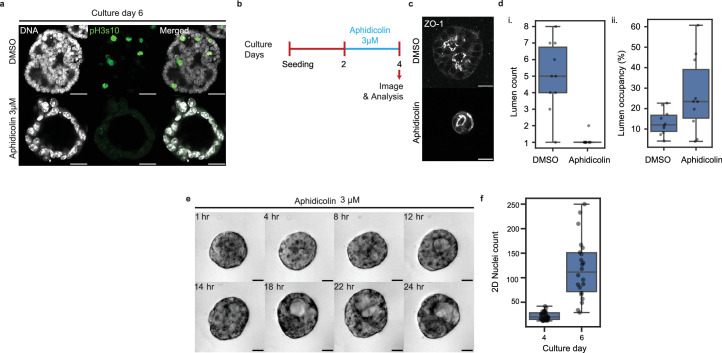
*In vitro* lumen morphogenesis under proliferation-arrested conditions. (**a)** Immunofluorescence images of branching organoids treated with DMSO or aphidicolin, stained for phospho-Histone 3 serine 10 (pH3s10, green) and DNA (white). Scale bar = 30 µm. **(b)** Schematic showing the experimental design of the aphidicolin assay. **(c)** Immunofluorescence images of DMSO- and aphidicolin-treated branching organoids on day 4, stained for ZO-1. Scale bar = 20 µm. **(d)** 2D quantification of lumen number (i) and lumen occupancy (ii) per organoid of DMSO- and aphidicolin-treated branching organoids on day 4. Boxplots (median, 25th–75th percentiles, and whiskers to 1.5× interquartile range) pooled from two independent experiments (n = 10 DMSO-treated and n = 10 aphidicolin-treated branching organoids). **(e)** Montage of live imaging of aphidicolin-treated branching organoids. Scale bar = 20 µm. **(f)** 2D quantification of the number of nuclei counted in the mid-plane of branching organoids on day 4 and day 6 of culture. Boxplot (median, 25th–75th percentiles, and whiskers to 1.5× interquartile range) pooled from three independent experiments (n = 35 branching organoids at day 4; n = 22 branching organoids at day 6). Source data

Given that our quantitative comparisons for cell proliferation rates and differential lumen pressure involved organoids grown in different culture media, we first aimed to specifically perturb cell proliferation in the same organoid medium. To test the model predictions, we used aphidicolin, which slows down or stops proliferation by inhibiting DNA polymerase A, thus arresting cells in the S-phase of the cell cycle. Lumen morphogenesis occurs over long timescales (days); accordingly, short-term (10-h) aphidicolin treatment had no detectable impact on intestinal organoid morphology^11^. We therefore treated branching organoids, the more proliferative subtype, at two stages: an early growth phase (day 2 to day 4), when lumen number increases due to de novo formation (Fig. 1c), and a late growth phase (day 4 to day 6), when lumen number decreases as connections form into a complex network (Fig. 1c,e). Under these treatment conditions, the cell cycle was arrested, leading to no or few organoids with phospho-histone-3 serine-10 (pH3s10)-positive cells (Extended Data Fig. 5a). We were unable to identify a dose of aphidicolin that slowed proliferation. During the early treatment with dimethylsulfoxide (DMSO) or aphidicolin (day 2–4; Extended Data Fig. 5b), branching organoids did not grow substantially in size, but lumen number decreased while lumen occupancy increased (Extended Data Fig. 5c,d). We then treated branching organoids with DMSO or aphidicolin at day 4, when multiple narrow lumina were already present, and analysed them at day 6 (Fig. 3a).

By day 6, aphidicolin-treated branching organoids exhibited a more spherical geometry, fewer lumina and higher lumen occupancy compared with DMSO-treated branching organoids (Fig. 3b,c and Extended Data Fig. 5e). These results indicate that stopping proliferation has a drastic impact on lumen morphology in the branching organoids. To mimic proliferation arrest in the in silico model, we conducted new simulations at the determined value of *ξ* = 0.12 and *τ*_V_ = 40, where cell cycles were halted once the organoids reached a cell number comparable to the average quantified in two dimensions on day 4, before aphidicolin treatment (Fig. 3d,e and Extended Data Fig. 5f). Moreover, we compared experimental organoids with numerical model phenotypes. Under these conditions, the in silico organoids evolved into spheres with single large spherical lumina, mirroring the lumen occupancy and numbers (Fig. 3f,g and Supplementary Videos 2 and 3) observed in experiments (Fig. 3h,i). This model shows that proliferation arrest leads to fusions of lumina over time and evolution to a single spherical lumen (Fig. 3d,e and Extended Data Fig. 5b). The fast proliferation of branching organoids is thus crucial to keep the system out of equilibrium and prevent relaxation of lumina to a single lumen via a slow fusion process.

### Controlling organoid lumen morphology through manipulation of osmotic pressure

Numerous studies theoretically predict or underscore lumen pressure’s influential role in determining organoid morphologies^9,20,25^. However, direct perturbation of lumen osmotic pressure was rarely combined with measurements of the effect on hydrostatic pressure and an assessment of the impact on complex lumen morphologies.

**Extended Data Fig. 6:**
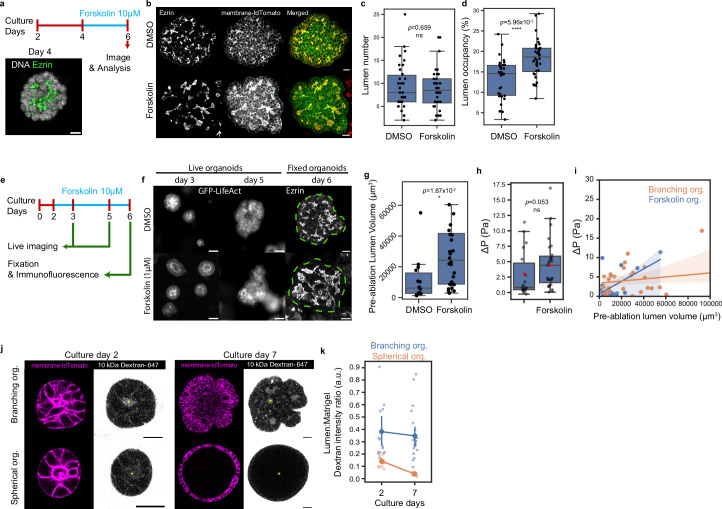
Lumen hydrostatic pressure inference of branching organoids under forskolin treatment and organoid permeability. **(a)** Schematic showing the experimental design of forskolin treatment from day 4 to day 6. Scale bar = 10 µm. **(b)** Immunofluorescence images of branching organoids expressing membrane-tdTomato and stained for Ezrin under DMSO and forskolin treatment at day 6. Scale bar = 20 µm. **(c)** 2D quantification of lumen number per organoid of DMSO- and forskolin-treated branching organoids on day 6. Boxplot (median, 25th–75th percentiles, and whiskers to 1.5× interquartile range) pooled from three independent experiments (n = 35 DMSO-treated and n = 40 forskolin-treated branching organoids). **(d)** 2D quantification of lumen occupancy per organoid of DMSO- and forskolin-treated branching organoids on day 6. Boxplot (median, 25th–75th percentiles, and whiskers to 1.5× interquartile range) of data pooled from three independent experiments (n = 27 DMSO-treated and n = 31 forskolin-treated branching organoids). **(e)** Schematic of experimental design for forskolin treatment from day 2 to day 6 and monitoring lumen morphogenesis during growth (day 3, 5, and 6). **(f)** Live images of branching organoids expressing GFP-LifeAct at day 3 and day 5 under DMSO and forskolin (1 µM) treatment, and immunofluorescence images at day 6 stained for Ezrin. Dashed green lines outline organoid surfaces. Scale bar = 10 µm. **(g)** 3D quantification of lumen volume in branching organoids treated with DMSO and forskolin before laser ablation. Boxplot (median, 25th–75th percentiles, and whiskers to 1.5× interquartile range). Data pooled from three independent experiments (n = 20 DMSO-treated and n = 39 forskolin-treated branching organoids). **(h)** Inference of lumen hydrostatic pressure (*∆P*) of DMSO- and forskolin-treated branching organoid lumina on day 6 of culture. Red dots indicate mean values (DMSO = 2.89 Pa; forskolin = 4.58 Pa). Boxplot (median, 25th–75th percentiles, and whiskers to 1.5× interquartile range) of data pooled from three independent experiments (n = 20 DMSO-treated and n = 39 forskolin-treated branching organoids). **(i)** Quantification showing the relationship between lumen hydrostatic pressure and pre-ablation lumen volume of branching organoids under DMSO and forskolin treatment. Linear-fit plot with ±95% confidence interval. Data pooled from three independent experiments (n = 20 DMSO-treated and n = 39 forskolin-treated branching organoids). **(j)** Live images of spherical and branching organoids expressing membrane-tdTomato treated with 10 kDa Dextran-647 at day 2 and day 7 of culture. Scale bar = 20 µm. **(k)** Quantification of luminal dextran levels (normalized to dextran levels in the Matrigel) in branching and spherical organoids at day 2 and day 7 of culture. Mean ± 95% confidence interval, pooled from three independent experiments (n = 12 and n = 24 branching organoids; n = 16 and n = 7 spherical organoids for day 2 and day 7, respectively). *P* values were determined by two-sided Mann-Whitney test (**c, d, g, h**). Source data

To perturb lumen osmotic pressure, we treated the branching organoids with forskolin, an activator of cystic fibrosis transmembrane conductance regulator (CFTR) ion channels, which triggers the secretion of chloride ions and bicarbonate into the lumen. The secretion of ions is expected to increase lumen osmotic pressure^9,20^. Forskolin treatment from day 4 to 6 in our branching organoids resulted in inflated lumina and increased lumen occupancy but did not yield a single spherical lumen (Extended Data Fig. 6a–d). As the lumen morphology is already committed to a complex network by this late growth phase (day 4–6), we instead applied forskolin from day 2, when each lumen is still individualized (Fig. 1c).

Although early treatment also enlarged the lumina, inflating them via forskolin at day 2 still did not redirect development towards a spherical lumen (Extended Data Fig. 6e,f). Although chloride ion secretion may transiently increase the lumen osmotic pressure and subsequent water influx, we conclude that it results in a change of lumen volume but does not lead to a sustained increase in lumen hydrostatic pressure in branching organoids (mean lumen hydrostatic pressures: DMSO, 2.9 Pa; forskolin, 4.6 Pa) (Extended Data Fig. 6g–i). Given the unexpected lack of lumen pressure increase by forskolin, we hypothesized that the epithelium of branching organoids may not retain solutes and water sufficiently to enable pressure increase.

### Epithelial permeability contributes to the regulation of lumen morphology

**Fig. 4:**
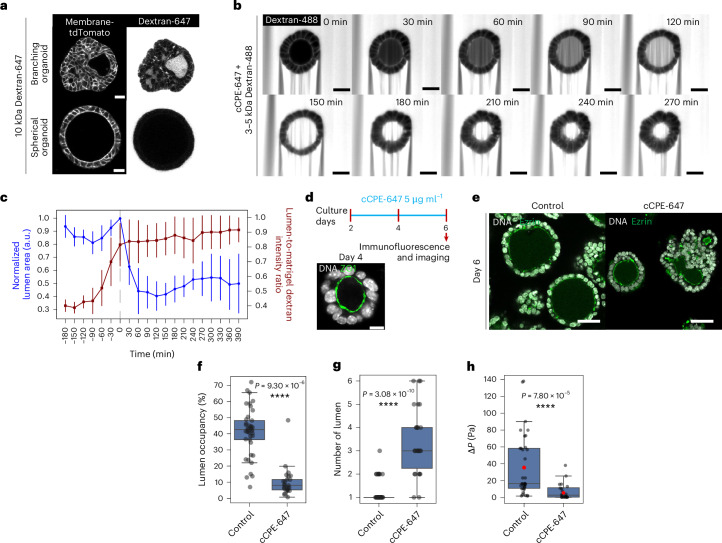
Transformation of spherical organoids into branching organoids through induced permeation. **a**, Live images of membrane-tdTomato-expressing branching and spherical organoids treated with 10 kDa Dextran-647, 3 h post treatment. Scale bar, 20 µm. **b**, A montage of live spherical organoids cotreated with 3–5 kDa Dextran-488 and cCPE-647 at various timepoints of treatment. Scale bar, 20 µm. **c**, A quantification of normalized levels of 3–5 kDa Dextran-488 in the lumen (lumen-to-Matrigel intensity ratio of 3–5 kDa Dextran-488) and normalized 2D lumen area (normalized to the lumen area at the timepoint before collapse) at various timepoints of cCPE-647 treatment. The data are presented as the mean ± standard deviation pooled from two independent experiments (*n* = 12 spherical organoids). **d**, A schematic showing the experimental design of cCPE-647 treatment assay. Scale bar, 10 µm. **e**, The immunofluorescence images of control and cCPE-647–treated spherical organoids on day 6. Scale bar, 30 µm. **f**,**g**, A 3D quantification of lumen occupancy (**f**) and lumen number (**g**) of control and cCPE-647-treated spherical organoids on day 6. The data are represented as boxplots showing the median (centre line), the 25th and 75th percentiles (box edges) and whiskers extending to 1.5× the interquartile range. The data are pooled from three independent experiments (*n* = 41 control; *n* = 26 cCPE-647 treatment). **h**, The inference of lumen hydrostatic pressure (∆*P*) of control and cCPE-647-treated spherical organoid lumina on day 6 of culture. The data are represented as boxplots showing the median (centre line), the 25th and 75th percentiles (box edges) and whiskers extending to 1.5× the interquartile range. The red dots indicate the mean values (control, 36.8 Pa; cCPE-647, 7.6 Pa). The data are pooled from two and three independent experiments for control and cCPE-647-treated conditions, respectively (*n* = 34 control; *n* = 20 cCPE-647 treatment). The *P* values were determined by a two-sided Mann–Whitney test for **f**, **g** and **h**. Source data

Epithelial paracellular permeability (or ‘tightness’) regulates the epithelial barrier function against ions, solutes and molecules^32^. To test permeability, we added 10 kDa fluorescent Dextran-647 into the culture media and monitored its appearance inside the lumen. Consistent with reports on MDCK cells and certain intestinal organoids, spherical organoids remained largely impermeable; after 3 h, they showed little-to-no luminal Dextran-647 signal, confirming a tight epithelial monolayer^9,33^. By contrast, branching organoids were consistently permeable, displaying both luminal Dextran-647 accumulation and fluorescence along paracellular spaces (Fig. 4a). These observations of epithelial permeability were already evident as early as culture day 2 and persisted through day 7 (Extended Data Fig. 6j,k), underscoring a stable, architecture-linked difference in tight-junction function. These experiments therefore reveal a striking divergence in epithelial permeability between branching and spherical organoids.

**Extended Data Fig. 7:**
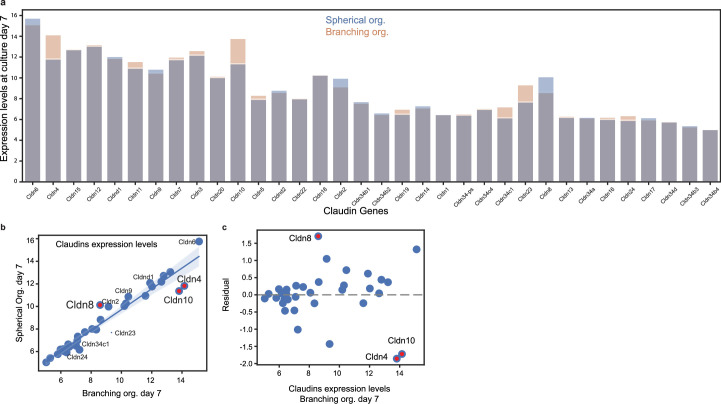
Claudin expression in branching and spherical organoids. **(a)** Microarray transcriptomics data showing claudin expression levels (log_2_) in branching organoids (blue) and spherical organoids (orange) on day 7 of culture. **(b)** Scatter plot with linear regression (±95% confidence interval) showing the relationship between claudin gene expression (log2) in branching and spherical organoids on day 7 of culture. Top 10 genes deviating from the regression are annotated, with red points marking the top 3 deviating genes. **(c)** Scatter plot showing the residuals of the linear regression model (from **b**). The gray dashed horizontal line at y = 0 indicates where residuals would lie if there were no deviation from the model. Source data

**Extended Data Fig. 8:**
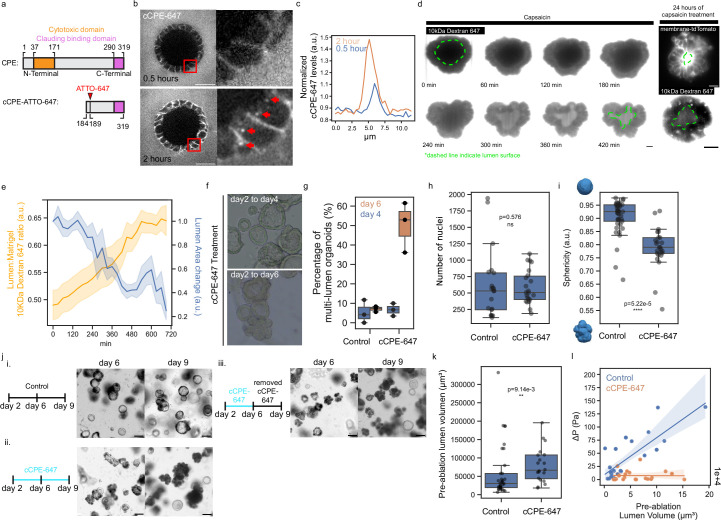
Lumen morphogenesis under permeabilization with cCPE-647 and capsaicin. **(a)** Schematic diagrams showing the full CPE (top) and truncated cCPE (bottom) linear structures, their cytotoxic and claudin-binding domains, and the ATTO-647 label site. **(b)** Live images of day 4 spherical organoids treated with cCPE-647 at 0.5 hours and 2 hours of incubation. Fluorescent signals indicate cCPE-647 in the Matrigel and paracellular spaces; red arrowheads mark cytoplasmic cCPE-647 puncta. Scale bar = 20 µm. **(c)** Line-profile quantification of cCPE-647 (normalized to Matrigel signal) across paracellular spaces at various time points of treatment. **(d)** Left: Montage of live spherical organoids co-treated with Dextran 10 kDa-647 and 100 µM capsaicin at various time points of treatment. Right: Live images of different spherical organoids after 24 hours capsaicin-treatment; expressing membrane-tdTomato (top) and 10 kDa Dextran-647 co-treated (bottom). Scale bar = 10 µm. **(e)** Quantification of normalized levels of 10 kDa Dextran-647 in the lumen (lumen-to-Matrigel intensity ratios) and the mean normalized 2D lumen area (normalized to the lumen area at the timepoint before collapse) at various time points of capsaicin treatment. Data are presented as mean ± 95% confidence interval, pooled from two independent experiments (n = 12 spherical organoids). **(f)** Phase-contrast images of spherical organoids treated with cCPE-647 from day 2 to day 4 (top) and from day 2 to day 6 (bottom) of culture. **(g)** Quantification of the percentage of organoids showing a multi-lumen phenotype in control and cCPE-647-treated spherical organoids from day 2 and analyzed at day 4 and 6. Boxplot (median, 25th–75th percentiles, and whiskers showing minimum and maximum values) pooled from three independent experiments. **(h**) 3D quantification of the number of nuclei in control and cCPE-647-treated spherical organoids on culture day 6. Boxplot (median, 25th–75th percentiles, and whiskers to 1.5× interquartile range) pooled from two and three independent experiments (n = 21 control; n = 22 cCPE-647) for control and cCPE-treated conditions, respectively. **(i)** 3D quantification of sphericity in control and cCPE-647-treated spherical organoids on culture day 6. Boxplot (median, 25th–75th percentiles, and whiskers to 1.5× interquartile range). Data pooled from three independent experiments (n = 41 control; n = 25 cCPE-647). **(j)** Schematic of experimental design and representative phase-contrast images of live spherical organoids at day 6 and day 9 under three conditions: (i) untreated, (ii) continuous cCPE-647 treatment from day 2 to day 9, (iii) cCPE-647 treatment from day 0 to day 6 followed by washout from day 6 to day 9. Scale bar = 40 µm. **(k)** Quantification of pre-ablation lumen volume of spherical organoids under control and cCPE-647 treatment conditions. Boxplot (median, 25th–75th percentiles, and whiskers to 1.5× interquartile range) pooled from three independent experiments (n = 34 control; n = 20 cCPE-647). **(l)** Quantification showing the relationship between lumen hydrostatic pressure and pre-ablation lumen volume of spherical organoids under control and cCPE-647 treatment. Linear-fit plot with ±95% confidence interval error bars of data pooled from three independent experiments (n = 34 control; n = 20 cCPE-647). *P* values were determined by two-sided Mann-Whitney test (**h, i, k**). Source data

To investigate whether increased permeability leads to decreased pressure and a subsequent change in lumen morphology, we used *Clostridium perfringens* enterotoxin (CPE), which forms pores in the epithelium and disrupts tight junctions by binding to claudins^34^^,35^. Reports show that CPE interacts most strongly with claudins 3 and 4 and with moderate-to-low affinity with claudins 1, 2, 6, 8, 9, 10 and 14 (refs. ^35–37^). Transcriptomic analysis confirms that both branching and spherical organoids express all of these claudins at prominent levels (Extended Data Fig. 7). In addition, non-cytotoxic recombinant forms of CPE (cCPE) have been utilized to manipulate barrier functions via tight-junction modulation^35,36^. Therefore, we synthesized cCPE labelled with ATTO-647 maleimide and used it to manipulate permeability and investigate alterations in lumen morphology in the spherical organoids (Extended Data Fig. 8a). Within 2 h of cCPE-647 treatment, signals were detected in the paracellular spaces of spherical organoids followed by cCPE-647 puncta in the cytoplasm, consistent with previous reports of internalization with claudin targets^38,39^ (Extended Data Fig. 8b,c). We then cotreated spherical organoids with 3–5 kDa Dextran-488 and cCPE-647 to assess permeability and monitor alterations in the lumen. The 3–5 kDa Dextran-488 signal inside the lumen rose steadily after CPE treatment, and once it plateaued, the luminal cross-sectional area dropped sharply, indicating a shrinkage of the lumen morphology due to increased permeability (Fig. 4b,c). As an alternative validation, spherical organoids were treated with capsaicin, which has been reported to permeabilize epithelial barriers by disrupting tight-junction integrity through altered cofilin phosphorylation and reduced occludin levels^40^. Although 24 h capsaicin treatment caused some toxicity in spherical organoids, short-term capsaicin treatment induced comparable luminal collapse (Extended Data Fig. 8d,e), reinforcing that epithelial barrier disruption is sufficient to drive lumen shrinkage through increased permeability.

To evaluate how sustained permeabilization impacts lumen morphogenesis, we cultured spherical organoids in standard medium supplemented with cCPE-647 from day 2 to 6 (Fig. 4d). Approximately half of the cCPE-647-treated spherical organoids converted to a branching morphology (Extended Data Fig. 8f,g), displaying diminished lumen occupancy and an increased number of individual lumina (Fig. 4e–g), while total cell number remained unchanged (Extended Data Fig. 8h). Consistent with these changes of the lumen, overall morphology of the spherical organoids under cCPE treatment are more branched (Extended Data Fig. 8i). Furthermore, when cCPE-647 was removed from the media after day 6, the spherical organoids did not revert to their spherical morphology (Extended Data Fig. 8j). Laser-ablation measurements confirmed that cCPE-647 reduced the lumen hydrostatic pressure, from a mean of 36.8 Pa in controls to 7.6 Pa in cCPE-647-treated spherical organoids (Fig. 4h), and abolished the positive correlation between pressure drop and lumen volume seen in untreated spheres (Extended Data Fig. 8k,l). Collectively, these experiments indicate that permeability has a key role in regulating lumen pressure with consequences on lumen morphology, independently of cellular proliferation.

### Dynamic epithelial permeability important for pancreatic ductal morphogenesis

**Fig. 5:**
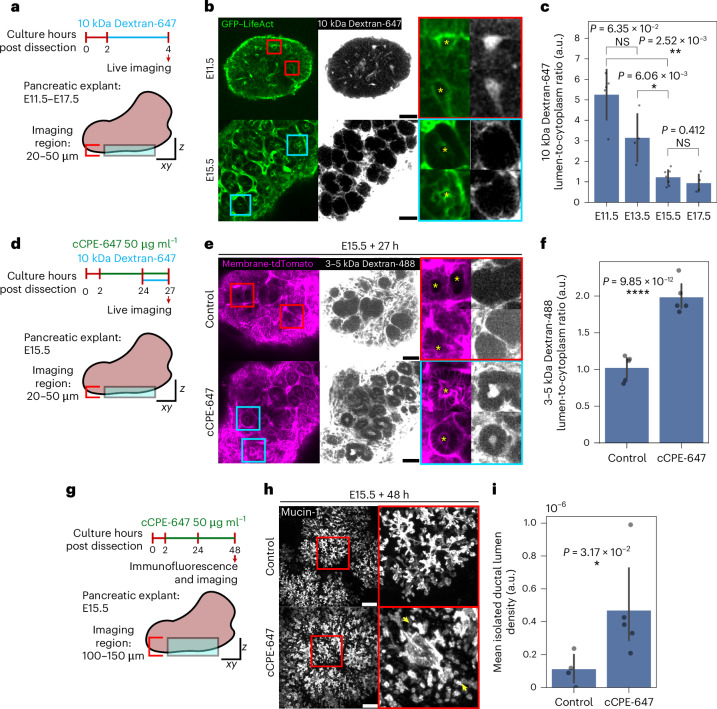
Epithelium permeability during pancreatic development and its impact on ductal morphogenesis. **a**, A schematic showing the experimental and imaging setup for the pancreatic explant permeability assay. **b**, Live images of GFP–LifeAct pancreatic explants (top, E11.5; bottom, E15.5) treated with 10 kDa Dextran-647. The yellow asterisks indicate lumina detected via GFP–LifeAct. Scale bar, 50 µm. **c**, A quantification of normalized levels of 10 kDa Dextran-647 (luminal dextran signal to neighbouring cytoplasmic dextran signal ratio, at mid-plane) in the lumen. The data are presented as the mean ± standard deviation, pooled from three independent experiments (*n* = 5 E11.5, *n* = 4 E13.5, *n* = 7 E15.5 and *n* = 4 E17.5 explant samples). **d**, A schematic showing the experimental and imaging setup for E15.5 pancreatic explants under control and cCPE-647 treatment for permeability assays. **e**, The live images of membrane-tdTomato-expressing E15.5 pancreatic explants under control and cCPE-647 treatment, treated with 3–5 kDa Dextran-488. Scale bar, 100 µm. **f**, A quantification of normalized levels of 3–5 kDa Dextran-488 (ductal/luminal dextran signal to neighbouring cytoplasmic dextran signal ratio) in the duct/lumen. The data are presented as the mean ± standard deviation, pooled from three independent experiments (*n* = 5 control and *n* = 5 cCPE-647-treated E15.5 explant samples). **g**, A schematic showing the experimental and imaging setup for E15.5 pancreatic explants under control and cCPE-647 treatment. **h**, The maximum-projected immunofluorescence images of control and cCPE-647-treated E15.5 pancreatic explants stained with Mucin-1, after 2 days of culture. The yellow arrowheads indicate isolated ductal lumina. Scale bar, 50 µm. **i**, A quantification of the mean isolated ductal lumen density (number of isolated lumina per volumetric tile; *x* = 88.5 µm, *y* = 88.5 µm, *z* = 24 µm). The data are presented as the mean ± standard deviation, pooled from three independent experiments (*n* = 4 control and *n* = 5 cCPE-647-treated E15.5 explant samples). The *P* values were determined by a two-sided Mann–Whitney test for **c**, **f** and **i**. NS, not significant. Source data

**Extended Data Fig. 9:**
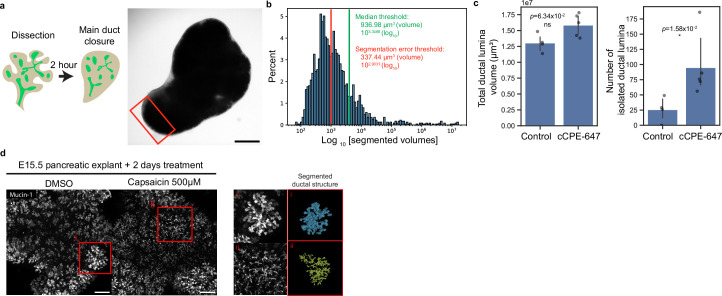
Lumen morphogenesis in pancreatic explants under cCPE-647 treatment. **(a)** Left: Schematic showing pancreas dissection and closure of the main duct during explant culturing. Right: Phase-contrast image of a pancreatic explant; the red box indicates the closed main duct region. Scale bar = 200 µm. **(b)** Histogram showing the distribution of segmented duct volumes (log scale). The red line and text indicate the threshold set to remove segmentation errors; the green line and text indicate the median value used to identify isolated lumina/duct. Data pooled from 2,271 segmented ductal volumes from 4 control and 5 cCPE-647–treated explants. **(c)** Left: Quantification showing the total volume of Mucin-1-positive ductal lumina per explant. Right: Quantification of the number of isolated ductal lumina per explant in control and cCPE-647–treated conditions. Mean ± standard deviation error bars of data pooled from 4 control and 5 cCPE-647–treated explants. **(d)** Left: Maximum-projected immunofluorescence images of E15.5 pancreatic explants stained with Mucin-1, after 2 days of DMSO and capsaicin (500 µM) treatment. Scale bar = 100 µm. Right: high-magnification image and 3D segmentation of ductal structures. *P* values were determined by two-sided Mann-Whitney test (**c**). Source data

As the permeability of organoids appeared to impact lumen morphology via its effect on pressure, we sought to investigate its in vivo relevance. We collected pancreases at E11.5, E13.5, E15.5 and E17.5 and incubated them with 10 kDa Dextran-647 to test epithelial permeability. Before adding dextran, the pancreatic explants were incubated for approximately 2 h in culture to allow the opened main duct (severed from the duodenum due to organ collection protocols) to close (Extended Data Fig. 9a). After 3 h of 10 kDa Dextran-647 incubation, we observed that the lumina of the E11.5 and E13.5 pancreases exhibited dextran signals. While at E15.5 and E17.5, explants had no luminal dextran signals (comparable to the cytoplasm), indicating that the epithelial permeability is dynamic during pancreatic development and that there is a transition from leaky to sealed epithelium around E15.5 (Fig. 5a–c).

As the treatment of spherical organoids with cCPE-647 resulted in a decrease of lumen hydrostatic pressure and the transformation of lumen morphology to be more branching organoid-like, we further investigated whether experimentally increasing permeability with cCPE-647 of E15.5 pancreas, a stage where the epithelium is tight, would also impact ductal morphology. After treating the E15.5 explants with cCPE-647, we performed the permeability assay and found that cCPE-647 is able to permeabilize the E15.5 pancreatic explants, as revealed by the presence of luminal dextran levels (Fig. 5d–f). To test the functional impact of epithelial permeabilization on ductal morphology, we treated E15.5 pancreatic explants with cCPE-647 for 48 h and stained for Mucin-1. Strikingly, cCPE-647 treatment resulted in numerous isolated ductal lumina (Fig. 5g–i). Unexpectedly, E15.5 explants treated with capsaicin (500 μM, 2 days) formed thinner ductal structures (Extended Data Fig. 9d), which we hypothesize reflects a combination of increased permeability and a potential contribution of altered cofilin phosphorylation impacting actin dynamics. These observations underscore that junctional permeability can modulate ductal morphogenesis in the developing pancreas, resembling our results in organoid systems.

## Discussion

While polarity establishment and apical membrane expansion via vesicle fusion are well-established drivers of lumen formation, our work uncovers how two critical factors, epithelial permeability-controlled lumen pressure and cell proliferation, cooperate to control lumen geometries in pancreatic organoids^41–46^. We demonstrate that the claudin-dependent permeability of the epithelium sets a low hydrostatic pressure that is conducive to complex lumen formation and that the balance between this pressure and the rate at which new apical surface is generated from cell duplication dictates whether lumina remain spherical or complex structures.

### Pressure as a morphogenetic cue

Computational studies predict that hydrostatic pressure arises from a balance of osmotic forces, fluid influx and paracellular leaks, thereby governing lumen growth and homeostasis^16–18,20,23,47^. Although quantitative lumen pressure measurements and estimations remain relatively scarce^9,21,26,28,29,48–51^, only a fraction of these studies have directly connected pressure heterogeneity to the molecular composition of tight junctions, particularly to complexes that jointly regulate junctional tension and paracellular leakiness^9^. Mukenhirn et al.^9^found that in ZO-1/2-deficient MDCK cysts, high cortical tension coupled with low pressure produces apical invaginations, resulting in lumen morphologies that are ‘flower shaped’. Although the lumen pressure we observe for spherical organoids is comparable to those previously reported^9,20,51^, the pressure in branching organoids is lower and enables the formation of the complex, narrow, interconnected lumina similar to those observed in the pancreas. We show that such a low pressure is due to epithelial leakiness and demonstrate that acute modulation of barrier function with the claudin-binding toxin cCPE alters hydrostatic pressure and reshapes lumen geometry.

### Claudin composition and combinatorial control of lumen pressure

Fluorescent dextran tracers report macromolecular leaks in the epithelium but not ion fluxes controlled by individual claudins. Because individual claudins possess distinct selectivities, their combined ‘claudin code’ sets the overall permeability profile of an epithelium^52,53^. Region-specific intestinal organoids illustrate this principle: differences in claudin composition translate into differential dextran penetration, whereas kidney and pancreas development show spatially segregated claudin patterns^33,54,55^. The ‘claudin code’ acts combinatorially, and other claudins may compensate for individual alterations in claudins. cCPE binds several claudins with distinct affinities; high for claudins 3 and 4 and moderate to low for claudins 1, 2, 6, 8, 9, 10 and 14 (refs. ^35–37^). As spherical organoids express higher levels of several of these targets (notably claudins 1, 2, 6 and 9, relative to the branching organoids), the ability of cCPE to decrease lumen pressure and alter lumen morphology probably reflects simultaneous removal of multiple barrier-forming isoforms^35–37^. Claudin 10 splice variant a (but not variant b) enhances Cl^−^ permeability in kidney cells; by analogy, claudin 10 (a and b) is the most highly expressed in branching organoids (Extended Data Fig. 7a–b), which may explain their lower lumen pressure and limited response to forskolin-induced CFTR activation^56,57^. Spherical organoids instead express less claudin 10 but more claudin 2 (Extended Data Fig. 7a,b), reminiscent of compensatory localization of claudin 2 in the proximal tubule of the kidney in claudin 10a-deficient mice^58^. The importance of claudin 2 is corroborated in adult pancreatic ductal organoids, as its knockout makes them morphologically unresponsive to forskolin even though they express high levels of CFTR^59^.

### Balancing pressure and cell proliferation rate

Another important parameter influencing lumen geometry is the cell proliferation rate, which dictates the rate at which nascent lumina appear at the end of cell division. In vitro, the FGFs and EGFs in the branching organoid medium promote fast cell division^13^. In vivo, the pre-acinar tips and acini have more proliferative capacity compared with the trunk region where progenitors reside^60,61^. As our branching organoids exhibit more acinar cells than spherical organoids, it is possible that their higher proliferation is also driven by an imbalance of cell types. Cerruti et al.^62^ have used cell packing patterns and modelling to evaluate how far from mechanical equilibrium MDCK cysts are. They further showed that this depends on their cell division rate and their cell rearrangement rate, with the latter being on a longer timescale than division^62^. Here, the geometric complexity of luminogenesis is captured by the multiphase-field model which systematically vary lumen osmotic pressure (*ξ*) and cell cycle timing (*τ*_V_), recreating the entire spectrum of observed organoid morphologies (from single spherical lumina to ramified, star-like structures)^25,63^. The impact of cell proliferation and lumen pressure on organoid morphologies has often been studied in isolation: cell cycle arrest blocks branch extension in pancreatic cancer organoids and forskolin-induced lumen inflation reshapes intestinal organoids^11,20,64,65^. Our work demonstrates that it is the joint tuning of these variables that determines final lumen geometry. We hypothesize that in branching organoids, rapid cell cycles supply lumen surface area faster than lumen volume (governed by osmotic pressure) can expand, biasing morphogenesis towards complex lumen geometries; in spherical organoids, slower proliferation allows pressure-driven lumen expansion to keep pace.

### A leaky to sealed epithelial transition in vivo

During pancreatic morphogenesis, epithelial polarity regulates lumen formation, fusion and maintenance^2,46^. The initially formed pancreatic ductal plexus network resolves into a tree by epithelial ‘loop closing’, and failures in lumen formation (as in Rab-11-knockout mice) result in discontinuous ducts^3,14,46,66^. We observe that the pancreatic epithelium is initially leaky and becomes impermeable from E15.5 onwards. Although our findings do not prove that the transition from a network to a tree is governed directly by lumen pressure after epithelium sealing, our organoid data make this an attractive possibility^14^. Because branching organoids remain permeable, they may model embryonic stages before E15.5. Our perturbation experiments show that prolonging permeability prevents proper lumen network connectivity. While this phenotype is not identical to that observed in spherical organoids, where a single spherical lumen transitions into multiple lumina upon permeability increase, it similarly results in the appearance of unconnected lumina. The milder phenotype in pancreatic explants probably reflects the higher complexity of the pre-existing ductal network at the time of perturbation compared with organoids. The molecular basis of the transition to a tight epithelium in vivo is currently unknown, but we can rule out that it results from the differentiation of endocrine or acinar cells, as both are present in branching and spherical organoids^13^. Moreover, branching organoids contain more acinar cells, yet acinar cells cannot be the source of leakiness since the in vivo epithelium becomes tight as acinar cell numbers increase between E14.5 and E18.5^61^ (Extended Data Fig. 1b–e). Definitively connecting permeability, lumen pressure and duct morphogenesis will require in vivo luminal pressure measurements with spatial resolution across tip and trunk domains to disentangle changes over time and space.

### Outlook

Our study employs a reductionist 3D culture model using primary cells freshly isolated from in vivo tissues, enabling experiments that would be challenging to perform in vivo. Despite its simplicity, this model system reveals that a finely-tuned balance between cell proliferation, lumen pressure and epithelial permeability dictates the morphological diversity observed in pancreatic organoids. Moreover, our findings reveal the crucial role of permeability in shaping the pancreatic ductal network. This work uncovers mechanisms that are potentially relevant to other organs exhibiting narrow interconnected ducts and to common cystic diseases affecting the pancreas as well as various branched organs. The system could, for example, be used to test the effect of drugs that revert disease phenotypes for possible therapeutic interventions.

## Methods

### Animals and permit

All experiments were performed in accordance with the German Animal Welfare Legislation (’Tierschutzgesetz’) after approval by the federal state authority Landesdirektion Sachsen (license DD24.15131/451/8). Mice were kept in standardized specific-pathogen-free conditions at the Biomedical Services Facility of the Max Planck Institute of Molecular Cell Biology and Genetics (MPI-CBG). The laboratory animal housing of the MPI-CBG is exclusively barrier housing. All mice are kept in individually ventilated cages under a 12 h–12 h light–dark cycle. The animal room temperature is maintained between 20 °C and 24 °C, and the relative humidity is 55% ± 10%. Both are subject to constant monitoring. In addition to Crl:CD1(ICR) (Charles River), genetically modified mouse lines LifeAct–EGFP^67^ and ROSAmT/mG^68^ were bred under C57BL/6N background (Janvier Labs).

### Pancreatic organoid culture

Mouse embryonic stage E10.5 was defined as noon of the day when the vaginal plug was detected in the mother. Pancreatic buds were dissected from E10.5 mice, and mesenchymal cells were removed using Tungsten needles^69^. To obtain cell aggregates, the buds were dissociated using TrypLE (12604013, Thermo Fisher Scientific) treatment for 12 min in a 37 °C incubator, followed by mechanical dissociation using pulled glass capillaries (BR708707, BRAND/Merck). The cell aggregates were seeded into 75% Matrigel (356231, Corning) in eight-well glass-bottom plates (80826, Ibidi) and left to polymerize at 37 °C in an incubator for 10 min. To grow branching organoids, a medium composed of 25 ng ml^−1^murine-FGF1 (450-33A, Perprotech), 25 ng ml^−1^murine-EGF (315-09, Perprotech), 2.5 U ml^−1^heparin (7980, Stemcell Technologies), 10 µM Y-27632 -dihydrochloride ROCK inhibitor (Y0503, Sigma-Aldrich), 16 nM phorbol-12-myristate-13-acetate (524400, Milipore), 100 ng ml^−1^murine-FGF10 (450-61, Perprotech), 500 ng ml^−1^murine-spondin-1 (315-32, Perprotech), 10% knockout serum replacement (10828-028, Gibco), 1% penicillin–streptomycin (15140-122, Sigma-Aldrich) and DMEM/F12 (1:1) 1× (+)ʟ-glutamine (11320-033, Gibco) was added. To grow spherical organoids, a medium composed of 64 ng ml^−1^murine-FGF2 (450-33, Peprotech), 10% B27 supplement (17504-044, Gibco), 10 µM Y-27632-dihydrochloride ROCK inhibitor (Y0503, Sigma-Aldrich), 1% penicillin–streptomycin (15140-122, Sigma-Aldrich) and DMEM/F12 (1:1) 1× (+)ʟ-glutamine (11320-033, Gibco) was added. The organoids were grown for 6 days in culture in an incubator at 37 °C 5% CO_2_. Medium exchange was carried out every 2 days.

### Pancreatic explant culture

Pancreatic buds were dissected from E11.5, E13.5, E15.5 and E17.5 mice. The mesentery was removed using Tungsten needles without removing mesenchymal cells^69^. To culture the pancreatic explants in suspension, the dissected buds were placed ibidis plates (80806, Ibidi) on a rocker inside the incubator at 37 °C 5% CO_2_. The explant media was composed of DMEM/F12 (1:1) 1× (+)ʟ-glutamine (11320-033, Gibco), 1% penicillin–streptomycin (15140-122, Sigma-Aldrich) and 10% FBS. Medium exchange was carried out every 2 days.

### Pharmacological and chemical treatment

To stop proliferation, branching organoids were treated with Aphidicolin (800153, Cell Signaling Technology/Merck), a DNA polymerase A inhibitor. Aphidicolin was used at a working concentration of 3 µM. To inflate the lumen, the CFTR activator Forskolin (32774, Tocris Bioscience) at was used 10 µM. To permeabilize the epithelium, spherical organoids were treated with capsaicin (2028, Sigma-Aldrich/Merck) at a working concentration of 100 µM for spherical organoids treatments and 500 µM for pancreatic explant treatments. To prevent epithelial branching of organoids, 2 MDa dextran (working concentration of 40 g l^−1^; Dextran T2000, Pharmacosmos) as added into the branching organoids media at culture day 4.

### Immunofluorescence

Pancreatic organoids were fixed with 4% formaldehyde (28908, Thermo Fisher Scientific) in PBS for 30 min at room temperature. Pancreatic explants were fixed in 4% formaldehyde for 2 h (stages E15.5 and onwards). The samples were blocked and permeabilized in 0.25% Triton (T8787, Sigma-Aldrich), 1% bovine serum albumin (BSA; A3059, Sigma-Aldrich) in PBS for 6 h at room temperature. The samples were incubated in primary antibody solution in 0.25% Triton, 1% BSA in PBS overnight at 4 °C and in secondary antibody solution in 0.25% Triton, 1% BSA in PBS overnight at 4 °C. To stain nuclei, Hoechst solution (34580, Invitrogen) or 4,6-diamidino-2-phenylindole (DAPI; ab228549, Abcam) was added in 0.25% Triton, 1% BSA in PBS for 4 h at room temperature after the incubation with the secondary antibody.

The primary antibodies used to mark the lumen were anti-Ezrin mouse (3C12) (sc-58758, Santa Cruz; dilution 1:400), anti-Mucin-1 hamster (MH1(CT2)) (MA5-11202, Thermo Fisher Scientific; dilution 1:400), anti-ZO-1(1A12) mouse (339100, Thermo Fisher Scientific; dilution 1:400), anti-aPKC (H1) mouse (sc-17781, Santa Cruz; dilution 1:400) and Alexa-488 Phalloidin (A12379, Thermo Fisher Scientific; dilution 1:1,000). To mark epithelial cells anti-Ecad (M108, Takara Bio; dilution 1:400) and anti-Sox9 (AB5535, Merck; dilution 1:400) were used. Antibody anti-Aurora B mouse (Becton Dickinson; dilution 1:400) was used to mark the abscission point (at the end of mitosis) and anti-phospho-histone-3 serine-10 mouse (3H10) (05-806, Millipore/Sigma-Aldrich; dilution 1:400) was used to mark dividing cells. Secondary antibodies used were goat anti-Armenian hamster IgG H&L (Alexa Fluor 568) (ab175716, Abcam; dilution 1:400), goat anti-Mouse IgG H&L (Alexa Fluor 488) preadsorbed (ab150117, Abcam; dilution 1:400) and anti-Mouse IgG H&L (Alexa Fluor 647) preadsorbed (ab150111, Abcam; dilution 1:400). Secondary antibodies, Alexa Fluor 488-, 568-, 594- and 647-conjugated (all from Invitrogen), were used at 1:400 dilution.

### CPE fragment expression, purification and labelling

#### Cloning

The non-cytotoxic, claudin-binding, C-terminal domain of CPE (Extended Data Fig. 8a) was expressed and purified according to Tachibana et al.^70^with few modifications. In details, a codon optimized cCPE fragment, amino acids 184–319 sequence (ERCVLTVPSTDIEKEILDLAAATERLNLTDALNSN PAGNLYDWRSSNSYPWTQKLNLHLTITATGQKYRILASKIVDFNIYSNNFNNLVKLEQSLGDGVKDHYVDISLDAGQYVLVMKA NSSYSGNYPYSILFQKF) (Twist Biosciences) was cloned into the p7XNH3 vector and tagged with a N-terminal 10xHis tag cleavable with human rhinovirus 3C protease^71^.

#### Expression

The *Escherichia coli* T7 Express strain (New Englad Biolabs) was transformed with p7XNH3-10xHis-3C-cCPE and pRare plasmids. A preculture was grown in lysogeny broth medium supplemented with 1% glucose, 30 µg ml^−1^kanamycin (kan) and 17 µg ml^−1^cloramphenicol (cm), overnight at 37 °C, with shaking at 150 rpm (Kunher shaker). *E. coli* cultures for induction were grown in Terrific broth, supplemented with 90 µg ml^−1^kanamycin and 17 µg ml^−1^chloramphenicol antibiotics at 37 °C. When the optical density at 600 nm reached a value of 0.6–0.8, the cultures were moved into a 18 °C shaking incubator. Protein expression was induced with 0.2 mM IPTG (Sigma), overnight at 18 °C.

#### Lysis, IMAC, His tag removal and size-exclusion purification steps

*E. coli* were collected by centrifugation at 6,000*g* for 10 min at 4 °C (JLA 8.1000 rotor, Beckman), lysed in lysis buffer (20 mM HEPES buffer, 0.5 M NaCl, 2 mM MgCl2, pH 7.2, 5% glycerol, 1 mM dithiothreitol) containing protease inhibitors EDTA-free (Bimake) and benzonase (Merck), with a high pressure homogenizer LM-20 (Microfluidics), using two passages at 20,000 psi. Insoluble material was removed by high-speed centrifugation (30,000*g*, 1 h, at 4 °C, in rotor JA12, Beckman) and by 0.45 µm filtration. immobilized metal affinity chromatography (IMAC) purification was performed with 5 ml HisTrap FF columns (Cytiva). After equilibration and loading, the column was washed with lysis buffer, supplemented with 20 mM and then 50 mM imidazole. Finally, 10xHis-tagged-cCPE was eluted with IMAC elution buffer (20 mM HEPES, 0.5 M NaCl, pH 7.2, 5% glycerol, 500 mM imidazole, 0.5 mM tris(2-carboxyethyl)phosphine (TCEP)). To remove the 10xHis tag, the protein was incubated with HRV3C protease (Merck) while dialysed against a size-exclusion buffer (20 mM HEPES pH 7.2, 300 mM NaCl, 5% glycerol, 0.5 mM TCEP) overnight at 4 °C. Size-exclusion chromatography was performed with HiLoad Superdex200 column (Cytiva), equilibrated in 20 mM HEPES pH 7.2, 300 mM NaCl, 5% glycerol, 0.5 mM TCEP.

#### cCPE labelling with ATTO-647

cCPE was labelled with ATTO-647 maleimide (Sigma), according to the manufacturer’s protocol. Labelled cCPE was separated from free dye excess using first a desalting nap-5 column (Cytiva), followed by gel filtration, using a 24 ml Superdex75 column (Cytiva) equilibrated in 20 mM HEPES pH 7.2, 300 mM NaCl, 5% glycerol, 0.5 mM TCEP. cCPE-labelled fractions were analysed by SDS–polyacrylamide gel electrophoresis. Positive fractions were pooled and protein concentrated. The degree of labelling was calculated to be 0.6.

### Viscosity estimation with 3D particle tracking

To estimate viscosity in approximately 90% H_2_O and ~1% glycerol, carboxyl fluorescent pink particles (CF-2058-2, Spherotech) were used at a 1:10 dilution to validate the viscosity measurement method. To estimate viscosity in the lumen, spherical organoids were treated with CellMask-orange (C10045, Invitrogen) at a concentration of 10 µg ml^−1^before image acquisition (Extended Data Fig. 4b). Following approximately 2 h of treatment, CellMask-orange-positive particles were imaged in a spinning-disk microscope. A 3D stack imaging was performed with a *z*-step size of 0.25–0.35 µm and a temporal resolution of 0.7–1.2 s per timepoint, depending on the total stack size, using the spinning-disk confocal microscope (see microscope details below).

To obtain particle segmentation and trajectories, images were denoised by using Noise2Void^72^. Afterwards, particles were manually cropped in three dimensions and time. To segment the particles, a combination of StarDist and accelerated pixel and object classification (APOC) Python-based tools were used to obtain 3D labels in time with manual corrections performed via Napari^73,74^. To obtain particle trajectories, LapTrack and Napari-Laptrack were used to track particles according to their distance and image-based features (intensities and size) between timeframes^75^(Extended Data Fig. 3b,c).

For the estimate of the diffusion coefficients of the particles, trackpy was utilized to obtain 3D mean squared displacement curves from the particle trajectories obtained^76^. To obtain accurate diffusion coefficients, particles with tracks longer than 20 timeframes and a regression of above 0.75 for the linear fit of the mean squared displacement were selected. To measure the size of particles, the biggest area (in the *z* axis of the segmentation) was used to derive the radius of the particle (Extended Data Fig. 3d,e). As a result, we observed that the particles had a negative correlation between particle radius and diffusion coefficients (Extended Data Fig. 3g). To estimate the viscosity of fluids using the obtained particle radius and diffusion coefficients, we used the Stokes–Einstein equation (Extended Data Fig. 3f). To validate this method to estimate fluid viscosity, all procedures were performed in approximately 90% H_2_O and approximately 1% glycerol (Extended Data Fig. 4j).

### Inference of hydrostatic lumen pressure with linear laser ablation

First, the organoids were taken out of the Matrigel to perform the laser ablation (Extended Data Fig. 4a). This was achieved by mechanically breaking the Matrigel dome (with organoids within) with pipettes followed by Liberase (CF-2058-2, Spherotech) incubation at 37 °C for 15 min to achieve enzymatic dissociation. Afterwards, individual organoids were transferred onto eight-well glass-bottom plates (80826, Ibidi) containing organogenesis medium with and without chemical treatments.

To create conduits accross the epithelium for inferring hydrostatic pressure of the lumen^9^, a laserablation was performed on day 6 organoids and spheres by utilizing a Zeiss LSM 780 NLO system (more details of microscope below) with a two-photon laser (titatnium/saphire). With the Zen Black software, the two-photon laser, with a power of 3.2 W at the laser head, was set to 100% laser power at a wavelength of 800 nm. A line scan with a width of 6.6–9.9 µm and 40–50-line repetitions across the epithelium at the middle plane of a lumen was performed to create a cut across the epithelium. Prior and post laser cutting, a 3D stack of the lumen was imaged (with a time resolution of 10-25 seconds: depending on the size of the lumen) to later measure the lumen volume changes.

FiJi software was used to quantify the image-based variables for the Hagen–Poiseulle model. The line-tool and measure functions were used to measure the monolayer thickness and smallest radius along single conduits (created by the laser ablation)^76,77^. LimeSeg, a Fiji plugin for the segmentation of 3D objects, was used to quantify the flow rate via lumen volume changes before and after the laser-ablation^78^.

### Epithelial permeability assay

As a readout for epithelial permeability of pancreatic organoids and explants, 3–5 kDa Dextran-Alex488 (D22914, Thermo Fisher Scientific) and 10 kDa Dextran-Alex647 (D22914, Thermo Fisher Scientific) were supplemented to the organoid and explant media. After 1–3 h of incubation, pancreatic organoids and explants were imaged with either a confocal microscope or light-sheet microscope.

### Transcriptome analysis

Branching and spherical organoids at day 7 of culture were lysed with lysis buffer RLT, and the RNA was purified following the manufacturer’s instructions (RNeasy Plus Micro Kit, 74034, Qiagen). The quality of the purified RNA using an Agilent 2100 Bioanalyzer, following the instructions of the manufacturer (Agilent RNA 6000 Pico Kit 5067–1513). Amplification of the extracted RNA (700 pg) was performed by Ovation Pico SL WTA system V2 (3312–48, Nugen). The samples were labelled with SureTag DNA labelling kit (5190–3391, Agilent Technologies), run on SurePrint G3 Mouse Gene Exp v2 Array (G4852B, Agilent Technologies), and signals were read by a SureScan Microarray Scanner (Agilent Technologies).

### EdU incorporation assay

Organoids were incubated with 10 µM EdU (Click-iTPlus EdU Alexa Fluor 647; C10640, Invitrogen) in organogenesis medium for 2 h at 37 °C and 5% CO_2_. Then, organoids were processed for immunostaining as described above. Permeabilization, blocking and Click-iT reaction for EdU detection were performed according to the manufacturer’s instructions.

### Microscopy

#### Spinning-disk microscopy

For live imaging and particle tracking experiments, imaging was performed using an Andor Revolution spinning-disk confocal microscope (Olympus IX83 inverted stand) equipped with a Yokogawa CSU-W1 scan head and Borealis illumination system for uniform excitation. The setup included a stage-top Z-piezo (400 µm travel range) and an environmental chamber for temperature and CO_2_ control, maintained at 37 °C and 5% CO_2_ during live imaging. Fluorophores were excited using 488 nm, 561 nm and 647 nm lasers. The reflector revolver was set to positions 3 (GFP), 4 (RFP) and 5 (CY5). Emission detection was configured using filter wheelS: LP 568, BP 525/50, BP 617/73 and BP 452/45. The images were acquired using Olympus U Plan SApo 30×/1.05 NA silicone and 40×/1.25 NA silicone objectives. The system was controlled using Andor iQ 3.6 software.

#### Single-photon and multiphoton confocal microscopy

For immunofluorescence imaging, the samples were imaged using a single-photon point-scanning confocal microscope (ZEISS LSM 700 Inverted) equipped with an Axio Observer.Z1 stand and a motorized stage. The system included two photomultiplier tubes (PMTs) and a transmission detector (T-PMT) for signal detection. Fluorophores were excited using laser diodes at 405, 488, 555 and 639 nm. The images were acquired using Zeiss Plan-Apochromat 20×/0.8 NA air and 25×/0.8 NA water/glycerol/oil objectives. The system was operated using Zeiss ZEN 2012 SP5 FP3 (black) software (64-bit version 14.0.25.201). For immunofluorescence imaging, live imaging and laser ablation, the samples were imaged using a ZEISS LSM 780 NLO 2-Photon Inverted confocal microscope equipped with a Zeiss Axio Observer.Z1 inverted stand. The system is equipped with a temperature- and CO_2_-controlled incubation chamber, maintained at 37 °C and 5% CO_2_ during live imaging and laser ablation. It supports combined singleand multiphoton imaging using a tunable pulsed near-infrared laser (Coherent Chameleon Vision II, 700–1,064 nm) for multiphoton excitation. Detection was achieved with two confocal PMTs, a 32-channel QUASAR GaAsP spectral detector, two transmitted-light PMTs (T-PMTs) and five non-descanned detectors for multiphoton imaging (including two GaAsP). The fluorophores were excited using laser diodes at 405, 488, 561 and 633 nm. The images were acquired using Zeiss Plan-Apochromat 20×/0.8 NA, LD LCI Plan-Apochromat 25×/0.8 NA oil/glycerol/water DIC and LD C-Apochromat 40×/1.1 NA water objectives. The system was controlled using Zeiss ZEN Black software (version 14.0.24.201).

#### Light-sheet microscopy

For organoid live imaging, images were acquired using an Viventis LS1 light-sheet microscope system (Leica) with inverted geometry. The system is equipped with dual-side illumination and adjustable light-sheet thickness and includes an environmental chamber maintaining a temperature of 37.5 °C and 5% CO_2_ with controlled humidity for long-term imaging. Illumination was provided by 488, 561, and 638 nm lasers, as well as transmitted light. Fluorescence was detected using GFP (525/50), GFP–mCherry and GFP–mCherry–iRFP filter sets. The images were captured with an Andor Zyla sCMOS camera (VSC-12371) using a Nikon Apo 25×/1.1 NA objective. The system was operated using Viventis Microscope Control software (version 2.0.0.2).

### Image analysis and quantification

#### 2D and 3D organoid and lumen segmentation and quantification

To segment the lumen and whole organoid structure, the images were first denoised using Noise2Void^72^. The images containing epithelial markers (nuclei and membranes) were summed using pyclesperanto-prototype^79^. The summed epithelium channel was then processed with Gaussian blur (sigma for *xyz* axes of 0.75–1.5) and Top-hat background removal (radius for *xyz* axes of 20–30 pixels). These processed channels, along with the lumen within the epithelium, were manually annotated using Napari to create training data for an APOC mode^73^. Using the trained APOC model, the epithelium channels were segmented. Inaccuracies in the prediction output were manually corrected with Napari or semi-automatically corrected using the binary processing functions of pyclesperanto-prototype. The lumen, identified as a 3D hole in the epithelium mask, and the segmentation output were used to generate triangulated meshes. To perform two-dimensional (2D) segmentation of organoids and lumen structures, the largest area along the *z* axis was selected from the 3D segmentation output for further analysis and quantifications.

To generate meshes from the lumen and epithelium, 3D Marching-Cube function of scikit-image were applied on the lumen binary and the epithelium binary that had been processed with the binary fill holes function of sciPy-image and rescaled pixel of pyclesperanto-prototype for isotropic pixels^79,80^. The generated meshes were smoothened using the Laplacian smoothening function of Trimesh^81^. Other features of the lumen and organoid mesh were obtained via Trimesh functions: integrated mean curvature, volume and surface area.

The following calculations were performed to obtain the morphological features of the lumen and organoids:

- Lumen and organoid sphericity: to numerically characterize the 3D morpholgoy of the objects we quantified the sphericity by applying the volume (*V*) and surface area (SA) obtained from the generated meshes (above) to the equation below^12^. This quantification resulted in perfect spheres exhibiting a sphericity values of 1 and in lower values with decreasing sphericity:
  $$\mathrm{Sphericity}=3\sqrt{4\pi V}/{\mathrm{SA}}^{3/2}$$
- The 2D and 3D lumen occupancy: to obtain the 3D lumen occupancy, volumes (*V*) obtained from the 3D segmentation of the lumen and organoid we used. For 2D lumen occupancy, lumen and organoid areas (*A*) from the mid-plane of organoids were used:

$$3{\rm{D}}\,{\rm{lumen}}\,{\rm{occupancy}}={V}_{{\rm{lumen}}}/{V}_{{\rm{organoid}}}$$

$$2{\rm{D}}\,{\rm{lumen}}\,{\rm{occupancy}}={A}_{{\rm{lumen}}}/{A}_{{\rm{organoid}}}$$

The lumen occupancy values are presented as percentages.

#### 3D lumen skeletonization and quantification

The segmented lumina (above) were skeletonized using the 3D skeletonization function in sciPy-image^80^. The output lumen skeleton binary images were further analysed using a skeleton analysis Python package Skan^82^.

#### 3D nuclei segmentation and quantification

The segmentation of nuclei in 3D images was performed using StarDist^74^. First, a subset of images with nuclei staining were manually annotated using Napari as training data to create a StarDist model. After, the trained model was applied to predict and segment the nuclei. The nuclear segmented output was used to quantify the number of EdU-, DAPI- and Hoescht-marked nuclei in organoids.

The following calculations were performed to obtain the proliferation features of the organoids:

- EdU-to-DNA ratio: to quantify active proliferation detected with the EdU incorporation assay we obtained the total number of EdU and DNA per organoid from the nuclear segmentation (above).
  From that we presented the data as a ratio
  $$\mathrm{EdU}:\mathrm{DNA}\,\mathrm{ratio}={\mathrm{count}}_{\mathrm{EdU}}/{\mathrm{count}}_{\mathrm{DNA}}$$
- Cleaved-caspase-3-to-DNA ratio: to quantify cell death population detected with the caspase 3 cleaved staining, we manually counted total number of cleaved-caspase-3-positive cells and DNA per organoid from the nuclear segmentation (above). From that we presented the data as a ratio
  $$\mathrm{Cleaved}\,\mathrm{caspase}\,3:\mathrm{DNA}\,\mathrm{ratio}={\mathrm{count}}_{\mathrm{cleaved}\,\mathrm{caspase}\,3}/{\mathrm{count}}_{\mathrm{DNA}}$$
- Doubling time: to quantify the rate of cell population doubling we obtained the average number

of cells at 48 h ($\bar{N}$_48_) and 96 h ($\bar{N}$_96_)

$$\mathrm{Doubling}\,\mathrm{time}\left(\mathrm{hours}\right)=\frac{\mathrm{Doubling}\left(\mathrm{hours}\right)\times \mathrm{ln}(2)}{\mathrm{ln}\left(\frac{{\bar{N}}_{96}}{{\bar{N}}_{48}}\right)}$$

#### Amylase population analysis

Nuclei were segmented with StarDist^74^. A subset of nuclei-stained images was manually annotated in Napari to train the StarDist model, which was then applied to the full data set. Each predicted nuclear label was dilated by four pixels to capture the cytoplasm, and the maximum voxel intensity in the amylase channel was recorded as punctate cytoplasmic localization of amylase makes the maximum more robust than the mean intensity per cell (Extended Data Fig. 1c). The values were normalized to the highest maximum intensity in each experiment to correct for staining and imaging variability.

To define high-, medium- and no/low-amylase levels per cell within an organoid, normalized maximum intensities (as mentioned above) was manually quantified using FiJi with visual inspection (Extended Data Fig. 1c, left). To establish a threshold between the two subpopulations, we estimated the probability density functions of the ‘high’ and ‘medium’ groups by fitting Gaussian kernel density estimators (KDEs) using the bandwidth selected via Silverman’s ‘rule-of-thumb’ (*h* ≈ 1.06 *σ* *n*^*−*1*/*5^)^83^. From the resulting KDE curves, we computed their difference across a fine grid of values and identified the first abscissa at which the sign of the difference changed (Extended Data Fig. 1d, right). This intersection point was adopted as the threshold separating the high- and medium-amylase levels per cell within an organoid.

#### Pancreatic duct segmentation and quantification

To segment the pancreatic ductal structures, the images were first denoised using Noise2Void^72^. These processed images were manually annotated using Napari to create training data for an APOC model^73^. Using the trained APOC model, the ductal structures were segmented.

The output segmentation/labels were further refined with pyclesperanto-prototype by (1) removing any labels touching the image edges, (2) removing labels smaller than 337 µm^3^ (apparent radius of 4.31 µm) in volume and (3) selecting smaller or isolated lumina by choosing labels below the median volume from the overall duct volume distribution^79^ (Extended Data Fig. 9b,c).

To quantify differences in the density of these isolated structures, a ‘virtual’ epithelium and duct region was generated by dilating, then eroding, the ductal labels by 50 pixels before merging them. Next, volumetric tiles of size 24 µm × 88.5 µm × 88.5 µm (*z*, *x*, *y*) were created, and those containing at least 50% volume overlap were selected for further analysis. Within each selected tile, the number of isolated lumen/duct labels was counted, and the resulting density was calculated by dividing that label count by the tile’s volume.

To quantify the level of dextran inside lumina/ducts, a minimum of two lumina/ducts per image was selected for mean intensity measurements. Meanwhile, for cytoplasmic dextran intensities, the neighbouring cells and the same number of intensities were taken. Using the rectangle tool in Fiji and measurement function, mean greyscale intensities were obtained. For the analysis, level of dextran in lumen were presented as a ratio between the lumen and cytoplasmic dextran intensity ratios. For data presentation, the mean level of dextran per explant were quantified.

### Phase-field model simulation

To computationally simulate the multicellular morphology, we applied the multicellular phase-field model^63^ with lumen phase^25,30^. Although the details of the model used in this study follow those in ref. ^25^, we changed the cell growth rule in the following way. In ref. ^25^, we assumed the term *α*[*V*_target_ − *V*_*i*_(*t*)] in the free energy functional with the constant target volume *V*_target_, as well as the variable area of each (*i*th) cell *V*_*i*_(*t*) and the constant prefactor *α*; by contrast, in this study, this term is replaced by *α*[*V*_target*,i*_(*t*) − *V*_*i*_(*t*)], where *V*_target*,i*_(*t*) is the time-dependent target volume, which allows us to control the cell growth rule. The time evolution of *V*_target*,i*_(*t*) was assumed to obey

1 $${\tau }_{V}\frac{{\rm{d}}{V}_{\mathrm{target},i}(t)}{{\rm{d}}t}=\bar{V}-{V}_{\mathrm{target},i}(t),$$

(*i.e*. exponential convergence towards the given constant $\bar{V}$ with the given characteristic convergence time *τ*_V_), so that we can control the typical cell growth duration by tuning the parameter *τ*_V_. Finally, in this paper, we do not assume the minimum duration for the division of each cell after the division of its mother cell, which we assumed in ref. ^25^. Parameter values used in this study are as follows; *α* = 1.0, $\bar{V}$ = 3.0, with *τ*_V_ = 1, 10, 20, 30, 40, 50, 60, 70, 80, 90 and *ξ* varying from 0.10 to 0.32 with increments of 0.02. In the model, at the end of cell division, micro-lumina are created at the middle point of the spindle poles (of a dividing cell) with a fixed size of value 0.7. The other parameter values and the initial conditions are set identical to those of ref. ^25^. To convert simulation units to physical units (see the table in ref. ^25^). The full set of the equations and parameter values used in this work are summarized in Supplementary Note.

### Statistics and reproducibility

Statistical analyses were performed using custom scripts in Python (v.3.11) with the SciPy statistics package (scipy.stats, v.1.13). Statistical significance was assessed using the two-sided Mann–Whitney test. No statistical method was used to predetermine sample size. As treatment with cCPE-647 in spherical organoids resulted in approximately 50% of the population displaying the multilumen phenotype (Extended Data Fig. 8e), analyses of lumen and branching morphologies, nuclear counts and lumen hydrostatic pressure were restricted to organoids exhibiting this phenotype. No data were excluded from the analyses. The experiments were not randomized, as groups were defined by experimental treatments. Explants and organoid cultures were randomly assigned to different treatment conditions. As data analyses were automated, investigators were not blinded to outcome assessment.

### Reporting summary

Further information on research design is available in the Nature Portfolio Reporting Summary linked to this article.

## Online content

Any methods, additional references, Nature Portfolio reporting summaries, source data, extended data, supplementary information, acknowledgements, peer review information; details of author contributions and competing interests; and statements of data and code availability are available at 10.1038/s41556-025-01832-5.

## Supplementary information

Supplementary Information Supplementary Note: details and descriptions of the multiphase-field model that was used, computation model (Section 1) and relationship between *ξ* and ∆*P* in the phase-field model (Section 2).

Reporting Summary

Supplementary Video 1 Cell and lumen dynamics in in silico organoid. Growth of in silico organoids τv = 20 and ξ = 0.26. Red regions represent the cells. Blue regions represent lumen.

Supplementary Video 2 Cell and lumen dynamics in in silico organoid with no stop in cell division. Growth of in silico organoids with τv = 40 and ξ = 0.12. Red regions represent the cells. Blue regions represent lumen.

Supplementary Video 3 Cell and lumen dynamics in in silico organoid with stop in cell division. Growth of in silico organoids with τv = 40 and ξ = 0.12. Cell division was stopped when in silico organoids reached 32 cell numbers. Red regions represent the cells. Blue regions represent lumen.

## Source data

Source Data Figs. 1–5 and Extended Data Figs. 1–9 All data are involved in one file, and each data file is named according to the relevant figure and extended data figure: 63 tabs, Figs. 1–5 and Extended Data Figs. 1–9.

## Data Availability

All other data supporting the findings of this study are available from the corresponding authors on reasonable request. Source data are provided with this paper.

## Code availability

Material and image analysis code requests should be addressed to the corresponding authors. The software code is available via GitHub at https://github.com/kana-fuji/MCPFM_tauV-model.git.
