# Supplementary material for: Permeability-driven pressure and cell proliferation control lumen morphogenesis in pancreatic organoids
Source: Nat Cell Biol. 2025 Dec 19;28(1):113–24. doi: 10.1038/s41556-025-01832-5 (PMC12807866; doi:10.1038/s41556-025-01832-5)
Supplement: Supplementary file 1 — Supplementary Note: details and descriptions of the multiphase-field model that was used, computation model (Section 1) and relationship between ξ and ∆P in the phase-field model (Section 2). [file 41556_2025_1832_MOESM1_ESM.pdf]

nature cell biology

Supplementary information

https://doi.org/10.1038/s41556-025-01832-5

Permeability-driven pressure and cell proliferation control lumen morphogenesis in pancreatic organoids

In the format provided by the authors and unedited

Supplementary Notes

0.1 Computational model

0.1.1 How to simulate the morphologies of organoids and lumens

In this Supplementary Note, we describe how we simulate the morphologies of organoids and lumens in this paper.

The key physical assumptions underlying the simulation dynamics are as follows:

1. All cells exhibit volume regulation, cell–cell adhesion, cortical tension (which is naturally introduced by the construction of the phase field model), and excluded volume effects, and all cells share identical properties.

2. The driving force for lumen growth—osmotic pressure—is constant over time and uniform across all lumens within each organoid. The hydrostatic pressure within the lumen is automatically determined by its balance with the tension in the surrounding cell layer in the model.

To implement this, we essentially adopted the mathematical framework known as the multi-cellular phase-field method, developed by Nonomura [1] and Akiyama et al. [2]. More specifically, the model used in this study was constructed based on the one we proposed in Tanida et al. [1], with a modification to the cell growth rule (see Materials and Methods in the main text or later in this section). The advantages of using phase-field methods for simulating organoids with lumens are discussed in Ref. [4].

0.1.2 Multicellular Phase Field Model

The organoid dynamics are simplified into two components: cells and lumens. The geometries of these components are represented by corresponding variables representing virtual fields, called phase fields. In this paper, the field variables $u_m(r,t)(m = 1, \cdots, M)$ and $s(r,t)$ are the phase fields and represent the shapes of individual cells and the lumen, respectively. Here, the index m denotes each cell, and M is the total number of cells. Regions where the phase-field variables take values near 1 and 0 correspond to the interior and exterior of each component, respectively.

The dynamics of the fields are based on the minimization of free energy via gradient descent. We followed the model construction principles described in Tanida et al. [3], with a newly introduced mechanism for controlling cell growth. The total free energy of the system is defined as follows:

$$E = E_u + E_s + E_{int}, \tag{1}$$

where E_u and E_s are the energy for cell and lumen, respectively, and E_{int} is the interaction energy. Each term is further decomposed into contributions from volume constraints, surface energy, osmotic pressure, and other effects:

$$\begin{aligned} E_u &= E_{pf}(u) + E_{surf}(u) + E_{vol}(u), & (2)\\ E_s &= E_{pf}(s) + E_{osm}(s), & (3)\\ E_{int} &= E_{adh} + E_{excl}. & (4) \end{aligned}$$

The part E_{pf} denotes the phase-field-specific energy terms, defined as:

$$E_{pf}(u) = \sum_m \int \Big[\frac{D_p}{2}|\nabla p|^2 + \frac{1}{4}p^2(1-p)^2\Big] d\boldsymbol{r}, \tag{5}$$

for the cell variable $p = u_m(m = 1, 2, \cdots, M)$ and lumen variable $p = s$. The coefficients $D_p \quad (p = u$ or $s)$ are positive diffusion constants and, in general, can be defined for u and s separately

as D_u and D_s. This double-well potential ensures that each field variable tends to take binary values (0 or 1), with an interface between regions. The surface energy of cells is given by

$$E_{surf} = \sum_m \frac{\gamma}{12} |h(u_m)|^2 d\boldsymbol{r}, \tag{6}$$

where γ is the surface tension of the cell. Hereafter, $h(\cdot)$ is a smooth function defined, on each phase field (p), as $h(p) \equiv p^2(3-2p)$. The osmotic pressure energy for the lumen is

$$E_{osm}(s) = -\frac{\xi}{6} V_l = -\frac{\xi}{6} \int h(s) d\boldsymbol{r}, \tag{7}$$

where ξ is the osmotic pressure of the lumen and V_l is the lumen volume given by $V_l = \int h(s) d\boldsymbol{r}$. Similarly, actual volume of i-th cell is defined by $V_i = \int h(u_i) d\boldsymbol{r}$. The adhesion energy defined as

$$E_{adh} = \sum_m^M \sum_{m'}^M \frac{\eta_u}{12} \int \nabla h(u_m) \cdot \nabla h(u_{m'}) d\boldsymbol{r} \tag{8}$$

where η_u is the cell-cell adhesion energy per area (length in 2D). The volume exclusion energy is given by

$$E_{excl} = \sum_m \Big[\sum_{m' \neq} \frac{\beta_u}{12} \int h(u_m) h(u_{m'}) d\boldsymbol{r} + \frac{\beta_{su}}{6} \int h(s) h(u_m) d\boldsymbol{r} \Big], \tag{9}$$

where β_u and β_{su} are coefficients for excluded volume interactions between cells and between cells and lumens, respectively. All these terms are the same as those in Tanida et al. [3].

The E_{vol} term represent the excess energy arising from deviations of the cell volume from its target value. They are defined as

$$E_{vol}(u) = \sum_m \frac{\alpha_u}{12} (V_{target,m}(t) - \int h(u_m) d\boldsymbol{r})^2, \tag{10}$$

where α_u is the positive constant representing bulk modulus. Unlike the model in Tanida et al. [3], here the target volume $V_{target,i}(t)$ is treated as a dynamic variable. Its time evolution is governed by Eq. (1) in the main text.

Assuming that each variable evolves by gradient descent of the free energy

$$\tau_u \frac{\partial u_m}{\partial t} = -\frac{\delta E}{\delta u_m}, \tau_s \frac{\partial s}{\partial t} = -\frac{\delta E}{\delta s} \tag{11}$$

the time evolution equations for each variable are obtained as follows:

$$\tau_u \frac{\partial u_m}{\partial t} = D_u \nabla^2 u_m + u_m(1-u_m)(u_m - \frac{1}{2} + f_u), \tag{12}$$

$$f_u = \alpha_u (V_m(t) - \int h(u_m) d\boldsymbol{r}) + \gamma_u \nabla^2 h(u_m) + \eta_u \nabla^2 \sum_{m' \neq m} h(u_{m'}) - \beta_u \sum_{m' \neq m} h(u_{m'}) - \beta_{su} h(s), \tag{13}$$

$$\tau_s \frac{\partial s}{\partial t} = D_s \nabla^2 s + s(1-s)(s - \frac{1}{2} + f_s), \tag{14}$$

$$f_s = -\beta_{su} \Psi_u + \xi. \tag{15}$$

Here, τ_u and τ_s are time constants for evolution.

The parameter values which we used in our simulations are as follows:
$\alpha = 1.0, \beta = 1.0, \beta_s = 1, 0, \gamma = 0.01, \eta = 0.008, D = 0.001, \tau = 1.0, D_s = 0.001, \tau_s = 1.0$ and $\bar{V} = 3.0$.
We simulated the cases for the various values of τ_V and ξ as the key control parameters:
$\tau_V = 1, 10, 20, 30, 40, 50, 60, 70, 80, 90$; ξ varying from 0.10 to 0.32 with increments of 0.02. At the end of cell division, micro-lumina are created at the middle point of the spindle poles (of a dividing cell) with a fixed size of value 0.7.

References

1. Makiko Nonomura, ”Study on Multicellular Systems Using a Phase Field Model.” PLOS ONE **7**(4):e33501 (2012). doi:10.1371/JOURNAL.PONE.0033501.

2. Masakazu Akiyama, Makiko Nonomura, Atsushi Tero and Ryo Kobayashi, ”Numerical study on spindle positioning using phase field method.” Physical Biology **16**(1):016005 (2018). doi:10.1088/1478-3975/AAEE45

3. Sakurako Tanida, Kana Fuji, Linjie Lu, Tristan Guyomar, Byung Ho Lee, Alf Honigmann, Anne Grapin-Botton, Daniel Riveline, Tetsuya Hiraiwa, Makiko Nonomura and Masaki Sano, ”Predicting organoid morphology through a phase field model: insights into cell division and lumenal pressure”, bioRxiv https://doi.org/10.1101/2024.04.22.590518.

4. Kana Fuji, Sakurako Tanida, Masaki Sano, Makiko Nonomura, Daniel Riveline, Hisao Honda and Tetsuya Hiraiwa, ”Computational approaches for simulating luminogenesis”, Seminars in Cell and Developmental Biology **131**, 173-185 (2022).

0.2 Relationship between ξ and ΔP in the phase field model

In the Phase Filed Model (PFM), the relationship between ξ and ΔP has been derived analytically in Tanida *et al.* from the growth dynamics of the domain in the two-dimensional (2D) case [1]. Considering the growth of the isolated lumen in one dimension first, the free energy associated with the lumen variable u_0 in one dimension is integrated by using the analytic solution of the interface, $u_0(x) = (1/2)(\tanh(x/(2\sqrt{2D})) + 1)$, as

$$F_0 = \int_\Omega \left[\frac{D}{2}|\nabla u_0|^2 + \frac{1}{4}u_0^2(1-u_0)^2 - \frac{\xi}{6}h(u_0)\right] \mathrm{d}\boldsymbol{r} = \frac{\sqrt{2D}}{12} - \frac{\xi}{6}V_l, \tag{16}$$

where D is the diffusion coefficient, $h(u) = u^2(3-2u)$, and $V_l = \int_\Omega h(u_0)\mathrm{d}\boldsymbol{r}$ is the volume of the lumen. The relation is extended to a 2D case and the growth of lumen volume V_l was derived from the kinetic equation based on the gradient flow of the free energy as,

$$\frac{dV_l}{dt} = -k\frac{\delta F_0}{\delta V_l} = \frac{k}{6}(\xi - \frac{T}{R}) = \frac{k}{6}(\xi - \Delta P) \tag{17}$$

where k is the kinetic constant, $T = \sqrt{D/2}$ is the tension of the interface, and $\frac{T}{R}$ can be regarded as the pressure difference, ΔP, between the inside and outside of the cyst in analogy to the lumen growth kinetics equation [2], This relation was confirmed successfully by numerical simulation.

The dynamics of lumen growth are described by the following equation [2],

$$\frac{dV_l}{dt} = \lambda_w A_{apical}(\Pi - \Delta P), \tag{18}$$

where V_l is the lumen volume, λ_w is the water permeability of the cell layer, A_{apical} is the apical area of the lumen surrounding the cell layer, Π is the osmotic pressure difference between the inside and outside of the cyst, ΔP is the hydrostatic pressure difference between the inside and outside. This relation can be written by using the lumen radius R for spherical lumen. Applying the relation $V_l = \pi R^2$ and $A_{apical} = 2\pi R$ in 2D, Eq. (2) becomes

$$\frac{dR}{dt} = \lambda_w(\Pi - \Delta P) = \lambda_w(\Pi - \frac{T}{R}), \tag{19}$$

where $T = \sqrt{D/2}$. For 3D case, employing $V_l = \frac{4\pi}{3}R^3$ and $A_{apical} = 4\pi R^2$ leads to

$$\frac{dR}{dt} = \lambda_w(\Pi - \Delta P) = \lambda_w\left[\Pi - T(\frac{1}{R} + \frac{1}{R})\right] = \lambda_w(\Pi - \frac{2T}{R}). \tag{20}$$

If a cell layer surrounds the lumen, the tension in the cell layer is expected to add to T. In such a case, total tension becomes $T_{total} = T + T_{cell}$, where T_{cell} denotes the tension by the cell layer.

To account for the effect of cell layer tension in PFM, a two-dimensional simulation of a cyst surrounded by a cell layer was performed with a parameter set and with the same rule in the simulation performed. By varying ξ, we obtain the time series of $V_l(t)$ (see Fig. S2(c). Assuming that the lumen is spherical (circular in 2D), we obtain $\frac{dR(t)}{dt} = \frac{1}{2\sqrt{\pi V(t)}}\frac{dV(t)}{dt}$ as a function of R for different values of ξ (Fig. S2 (d)). Next, the value of ΔP for each value of ξ can be estimated using the value of dR/dt obtained above,

$$\Delta P = \xi - \frac{1}{\lambda_w}\frac{dR(t)}{dt}|_{R=1}, \tag{21}$$

where $\frac{dR(t)}{dt}$ depends on the value of R, therefore we used the value at $R = 1$. Estimated ΔP values for various ξ are shown in Supplementary Figure 2(e). From the best fit in Supplementary Figure 2(e), we obtain $\Delta P = 0.68\xi - 0.0055$. The slope is slightly less than 1, with an offset corresponding to the presence of a critical osmotic pressure required for lumen growth.

Note that this is a two-dimensional case; in the three-dimensional case, the three-dimensional hydrostatic pressure difference, denoted by ΔP_3, is twice larger than the two-dimensional value for the same tension T. Thus, we employ the relation, $\Delta P_3 = 2\Delta P_2$, where ΔP_2 denotes the hydrostatic pressure difference in 2D. This yields an empirical relationship between ξ and ΔP in the 3-dimensional PFM, $\Delta P_3 = 2 \times (0.68\xi - 0.055) = 1.36\xi - 0.11$. In the Figure 2(ii), branched lumens are observed under low osmotic pressure conditions ($\xi_{org} = 0.14 \pm 0.02$), while spherical lumens are observed under high osmotic pressure conditions ($\xi_{sphere} = 0.30 \pm 0.02$). The ratio is $\xi_{sphere}/\xi_{org} \sim 2.1$. This ratio corresponds to the hydrostatic pressure ratio $\Delta P_{spare}/\Delta P_{org} = 2.11.36 \sim 2.9$. Compared to the hydrostatic pressure ratio observed in the experiment (3.4–7.0), this ratio in the simulation is slightly smaller, but the trend is consistent, and it is considered to be within the acceptable range of error for theoretical predictions.

References

1. S. Tanida, K. Fuji, L. Lu, T. Guyomar, B. H. Lee, A. Honigmann, A. Grapin-Botton, D. Riveline, T. Hiraiwa, M. Nonomura, and M. Sano, “Predicting organoid morphology through a phase field model: Insights into cell division and lumenal pressure,” PLOS Computational Biology, vol. 21, no. 8, pp. 1–37, 2025.

2. A. Torres-S´anchez, M. Kerr Winter, and G. Salbreux, “Tissue hydraulics: Physics of lumen formation and interaction,” Cells Development, vol. 168, p. 203724, 2021. Quantitative Cell and Developmental Biology.
