# Supplementary material for: Permeability-driven pressure and cell proliferation control lumen morphogenesis in pancreatic organoids
Source: Nat Cell Biol. 2025 Dec 19;28(1):113–24. doi: 10.1038/s41556-025-01832-5 (PMC12807866; doi:10.1038/s41556-025-01832-5)
Supplement: Supplementary file 2 — Reporting Summary [file 41556_2025_1832_MOESM2_ESM.pdf]

nature portfolio

Corresponding author(s): Anne Grapin-Botton

Last updated by author(s): 22/10/2025

Reporting Summary

Nature Portfolio wishes to improve the reproducibility of the work that we publish. This form provides structure for consistency and transparency in reporting. For further information on Nature Portfolio policies, see our Editorial Policies and the Editorial Policy Checklist.

Statistics

For all statistical analyses, confirm that the following items are present in the figure legend, table legend, main text, or Methods section.

n/a	Confirmed	
☐	☒	The exact sample size (*n*) for each experimental group/condition, given as a discrete number and unit of measurement
☐	☒	A statement on whether measurements were taken from distinct samples or whether the same sample was measured repeatedly
☐	☒	The statistical test(s) used AND whether they are one- or two-sided<br>*Only common tests should be described solely by name; describe more complex techniques in the Methods section.*
☒	☐	A description of all covariates tested
☐	☒	A description of any assumptions or corrections, such as tests of normality and adjustment for multiple comparisons
☐	☒	A full description of the statistical parameters including central tendency (e.g. means) or other basic estimates (e.g. regression coefficient) AND variation (e.g. standard deviation) or associated estimates of uncertainty (e.g. confidence intervals)
☐	☒	For null hypothesis testing, the test statistic (e.g. *F*, *t*, *r*) with confidence intervals, effect sizes, degrees of freedom and *P* value noted<br>*Give P values as exact values whenever suitable.*
☒	☐	For Bayesian analysis, information on the choice of priors and Markov chain Monte Carlo settings
☒	☐	For hierarchical and complex designs, identification of the appropriate level for tests and full reporting of outcomes
☒	☐	Estimates of effect sizes (e.g. Cohen's *d*, Pearson's *r*), indicating how they were calculated

Our web collection on statistics for biologists contains articles on many of the points above.

Software and code

Policy information about availability of computer code

Data collection

Pyrat (Scionics) was used to manage the mouse colony.
All spinning-disk microscope images were performed with a Olympus IX 83 inverted stand driven by the Andor iQ 3.6 software.
Laser-ablation and confocal imaging was performed by utilizing a Zeiss LSM 780 NLO system (Zen Black v11.00 software)
Light sheet imaging was performed by utilizing the Viventis Microscope system (Viventis microscope control (version 2.0.0.2))

Data analysis

2D & 3D organoid and lumen segmentation and quantificationTo segment the lumen and whole organoid structure, images were first denoised using Noise2Void6. Imagescontaining epithelial markers (nuclei and membranes) were summed using pyclesperanto-prototype14.The summed epithelium channel was then processed with Gaussian blur (sigma for xyz axes = 0.75-1.5)and Top-hat background removal (radius for xyz axes = 20-30). These processed channels, along withthe lumen within the epithelium, were manually annotated using Napari to create training data for anAPOC model7. Using the trained APOC model, the epithelium channels were segmented. Inaccuraciesin the prediction output were manually corrected with Napari or semi-automatically corrected usingthe binary processing functions of pyclesperanto-prototype. The lumen, identified as a 3D hole in theepithelium mask, and the segmentation output were used to generate triangulated meshes. To perform2D segmentation of organoids and lumen structures, the largest area along the z axis was selected fromthe 3D segmentation output for further analysis and quantifications.To generate meshes from the lumen and epithelium, 3D Marching-Cube function of scikit-imagewere applied on the lumen binary and the epithelium binary, that had been processed with the binaryfill holes function of scipy-image and rescaled pixel of pyclesperanto-prototype for isotropic pixels14, 15.The generated meshes were smoothened using the Laplacian smoothening fucntion of Trimesh16. Otherfeatures of the lumen and organoid meshes were obtained via Trimesh functions: integrated meancurvature, volume, and surface area.The following calculations were performed to obtain the morphological features of the lumen andorganoids:• Lumen and organoid sphericity: to numerically characterize the 3D morpholgoy of the objectswe quantified the reduced volume (υ) by applying the volume (V) and surface area (SA) obtainedfrom the generated meshes (above)17. This

quantification resulted in perfect spheres exhibiting areduced volume of 1 and in lower values with decreasing sphericity.Sphericity = 3√4π V / SA3/2
• 2D and 3D Lumen occupancy (LO): to obtain the 3D lumen occupancy, volumes (V) obtainedfrom the 3D segmentation of the lumen and organoid we used. For 2D lumen occupancy, lumenand organoid areas (A) from the mid-plane of organoids were used.3D Lumen occupancy = VLumen/VOrganoid2D Lumen occupancy = ALumen/AOrganoidThe lumen occupancy values are presented as percentages, except for Extended Data Figure 3.3D lumen skeletonization and quantificationThe segmentation out of the lumen (above) were skeletonized using the 3D skeletonization function inscipy-image15. The output lumen skeleton binary images were further analyzed using a skeleton analysispython package Skan18.3D nuclei segmentation and quantificationThe segmentation of nuclei in 3D-images was performed using StarDist8. First, a subset of images withnuclei staining were manually annotated using Napari as training data to create a StarDist model. After,the trained model was applied to predict and segment the nuclei. The nuclear segmented output wasused to quantify the number of EdU-, DAPI-, and Hoescht-marked nuclei in organoids.The following calculations were performed to obtain the proliferation features of the organoids:• EdU:DNA ratio: To quantify active proliferation detected with the EdU incorporation assay weobtained the total number of EdU and DNA per organoid from the nuclear segmentation (above).From that we presented the data as ratioEdU : DN A Ratio = CountEdU /CountDN A• Cleaved Caspase-3:DNA ratio: To quantify cell death population detected with the Caspase 3cleaved staining, we manually counted total number of Cleaved Caspase-3 positive cells and DNAper organoid from the nuclear segmentation (above). From that we presented the data as ratioCleaved − Caspase − 3 : DN A Ratio = CountCleaved−Caspase−3/CountDN A• Doubling time: To quantify the rate of cell population doubling we obtained the average numberof cells at 48 hours (N 48) and 96 hours(N 96)Doubling time(hours) = Duration(hours) × ln(2)ln N 96N 48Amylase population analysisNuclei were segmented with StarDist8. A subset of nuclei-stained images was manually annotated innapari to train the StarDist model, which was then applied to the full data set. Each predicted nuclearlabel was dilated by four pixels to capture the cytoplasm, and the maximum voxel intensity in theamylase channel was recorded as punctate cytoplasmic localisation of amylase makes the maximummore robust than the mean intensity per cell (Extended Data Figure 1c). Values were normalised to thehighest maximum intensity in each experiment to correct for staining and imaging variability.To define high-, medium-, and no/low-amylase levels per cell within an organoid, normalized maximumintensities (as mentioned above) was manually quantified using FiJi with visual inspection (ExtendedData Figure 1c: left). To establish a threshold between the two sub-populations, we estimated theprobability density functions of the "high" and "medium" groups by fitting Gaussian kernel density estimators (KDEs) using the bandwidth selected via Silverman's "rule-of-thumb" h ≈ 1.06 σ n−1/519.From the resulting KDE curves, we computed their difference across a fine grid of values and identifiedthe first abscissa at which the sign of the difference changed (Extended Data Figure 1d: right). Thisintersection point was adopted as the threshold separating the high- and medium-amylase levels per cellwithin an organoid.Pancreatic duct segmentation and quantificationTo segment the pancreatic ductal structures, images were first denoised using Noise2Void6. Theseprocessed images were manually annotated using Napari to create training data for an APOC model7.Using the trained APOC model, the ductal structures were segmented.The output segmentation/labels were further refined with pyclesperanto-prototype by (i) removingany labels touching the image edges, (ii) removing labels smaller than 337 µm3 (apparent radius of 4.31µm) in volume, and (iii) selecting smaller or isolated lumina by choosing labels below the median volumefrom the overall duct volume distribution (Extended Data Figure 9b - c)14.To quantify differences in the density of these isolated structures, a "virtual" epithelium and ductregion was generated by dilating, then eroding, the ductal labels by 50 pixels before merging them. Next,volumetric tiles of size 24µm × 88.5µm × 88.5µm (z, x, y) were created, and those containing at least50% volume overlap were selected for further analysis. Within each selected tile, the number of isolatedlumen/duct labels was counted, and the resulting density was calculated by dividing that label count bythe tile's volume.

For manuscripts utilizing custom algorithms or software that are central to the research but not yet described in published literature, software must be made available to editors and reviewers. We strongly encourage code deposition in a community repository (e.g. GitHub). See the Nature Portfolio guidelines for submitting code & software for further information.

Data

Policy information about availability of data

All manuscripts must include a data availability statement. This statement should provide the following information, where applicable:

- Accession codes, unique identifiers, or web links for publicly available datasets
- A description of any restrictions on data availability
- For clinical datasets or third party data, please ensure that the statement adheres to our policy

Material and image analysis code requests should be addressed to the corresponding author. The software code used for the phase field model simulation is available in the Github: https://github.com/kana-fuji/MCPFM_tauV-model.git

Research involving human participants, their data, or biological material

Policy information about studies with human participants or human data. See also policy information about sex, gender (identity/presentation), and sexual orientation and race, ethnicity and racism.

Reporting on sex and gender	*Use the terms sex (biological attribute) and gender (shaped by social and cultural circumstances) carefully in order to avoid confusing both terms. Indicate if findings apply to only one sex or gender; describe whether sex and gender were considered in study design; whether sex and/or gender was determined based on self-reporting or assigned and methods used. Provide in the source data disaggregated sex and gender data, where this information has been collected, and if consent has been obtained for sharing of individual-level data; provide overall numbers in this Reporting Summary. Please state if this information has not been collected. Report sex- and gender-based analyses where performed, justify reasons for lack of sex- and gender-based analysis.*
Reporting on race, ethnicity, or other socially relevant groupings	*Please specify the socially constructed or socially relevant categorization variable(s) used in your manuscript and explain why they were used. Please note that such variables should not be used as proxies for other socially constructed/relevant variables (for example, race or ethnicity should not be used as a proxy for socioeconomic status). Provide clear definitions of the relevant terms used, how they were provided (by the participants/respondents, the researchers, or third parties), and the method(s) used to classify people into the different categories (e.g. self-report, census or administrative data, social media data, etc.) Please provide details about how you controlled for confounding variables in your analyses.*

Population characteristics	*Describe the covariate-relevant population characteristics of the human research participants (e.g. age, genotypic information, past and current diagnosis and treatment categories). If you filled out the behavioural & social sciences study design questions and have nothing to add here, write "See above."*
Recruitment	*Describe how participants were recruited. Outline any potential self-selection bias or other biases that may be present and how these are likely to impact results.*
Ethics oversight	*Identify the organization(s) that approved the study protocol.*

Note that full information on the approval of the study protocol must also be provided in the manuscript.

Field-specific reporting

Please select the one below that is the best fit for your research. If you are not sure, read the appropriate sections before making your selection.

☒ Life sciences ☐ Behavioural & social sciences ☐ Ecological, evolutionary & environmental sciences

For a reference copy of the document with all sections, see nature.com/documents/nr-reporting-summary-flat.pdf

Life sciences study design

All studies must disclose on these points even when the disclosure is negative.

Sample size	No sample size pre-determination method. No statistical methods were used to pre-determine sample sizes but our sample sizes are similar or better than those reported in previous publications:<br><br>Yang et al., 2021:<br>https://www.nature.com/articles/s41556-021-00700-2<br><br>Lu et. al. 2025:<br>https://www.nature.com/articles/s41467-025-60780-8#Sec21<br><br>Greggio et. al 2013:<br>https://pubmed.ncbi.nlm.nih.gov/24130330/
Data exclusions	No data was excluded
Replication	The experiments were repeated at 2-5 times. and we indicate specific information in all legends. No experiment was excluded. All attempts at replication were successful.
Randomization	Samples were distributed randomly between treated-wells and non-treated wells.
Blinding	No blinding method was performed. The same investigator carried out the treatments and the analysis and therefore it was easy to remember in which well the treatment was made. However, the analysis was largely automated making the collection of numbers blind to the investigator.

Reporting for specific materials, systems and methods

We require information from authors about some types of materials, experimental systems and methods used in many studies. Here, indicate whether each material, system or method listed is relevant to your study. If you are not sure if a list item applies to your research, read the appropriate section before selecting a response.

Materials & experimental systems

n/a	Involved in the study	
☐	☒	Antibodies
☒	☐	Eukaryotic cell lines
☒	☐	Palaeontology and archaeology
☐	☒	Animals and other organisms
☒	☐	Clinical data
☒	☐	Dual use research of concern
☒	☐	Plants

Methods

n/a	Involved in the study	
☒	☐	ChIP-seq
☒	☐	Flow cytometry
☒	☐	MRI-based neuroimaging

Antibodies

Antibodies used	The primary antibodies used to mark the lumen were:

Antibodies used	- anti-Aurora B (Becton Dickinson),<br>- anti-Ezrin (3C12) (sc-58758, Santa Cruz)(dilution 1:400),<br>- anti-Mucin-1 (MH1(CT2)) (MA5-11202, Thermofisher Scientific)(dilution 1:400),<br>- anti-ZO1(1A12) (339100, ThermoFisher Scientific)(dilution 1:400),<br>- anti-aPKC (H1) (sc-17781, Santa Cruz)dilution 1:400),<br>- Alex-488 Phalloidin (A12379, Thermofisher Scientific)(dilution 1:1000),<br>- anti-Ecad (M108, TakaraBio)(dilution 1:400),<br>- anti-Sox9 (AB5535, Merk)(dilution 1:400),<br>- anti-phosho Histone-3 serine-10 (3H10) (05-806, Millipore/Sigma Aldrich)(dilution 1:400),<br><br>Secondary antibodies were:<br>- goat anti-Armenian hamster IgG H&L (Alexa Fluor 568) (ab175716, Abcam)(dilution 1:400),<br>- goat anti-Mouse IgG H&L (Alexa Fluor 488) preadsorbed (ab150117, Abcam)(dilution 1:400),<br>- anti-Mouse IgG H&L (Alexa Fluor 647) preadsorbed (ab150111, Abcam)(dilution 1:400).
Validation	The antibodies are all frequently used antibodies. We verified that the subcellular localization was the expected one: nuclear, absicion point, membranes, apical membranes

Animals and other research organisms

Policy information about studies involving animals; ARRIVE guidelines recommended for reporting animal research, and Sex and Gender in Research

Laboratory animals	Genetically modified mouse lines LifeAct-EGFP and ROSAmT/mG on a c57BL/6N background (Janvier Labs c57BL/6N source strain, then bred in our facility). The samples were embryos at E10.5. The laboratory animal housing of the MPI-CBG is exclusively barrier housing. All mice are kept in individually ventilated cages under a 12h:12h light:dark cycle. The animal room temperature is maintained between 20 and 24°C and the relative humidity is 55±10%. Both are subject to constant monitoring. Sterile food and water were given ad libitum.
Wild animals	*Provide details on animals observed in or captured in the field; report species and age where possible. Describe how animals were caught and transported and what happened to captive animals after the study (if killed, explain why and describe method; if released, say where and when) OR state that the study did not involve wild animals.*
Reporting on sex	The experiments were done on embryos. The embryos were not sexed and organoids originated from a mix of embryos and thus sex.
Field-collected samples	*For laboratory work with field-collected samples, describe all relevant parameters such as housing, maintenance, temperature, photoperiod and end-of-experiment protocol OR state that the study did not involve samples collected from the field.*
Ethics oversight	All experiments were performed in accordance with the German Animal Welfare Legislation ("Tierschutzgesetz") after approval by the federal state authority Landesdirektion Sachsen (license DD24.1- 5131/451/8).

Note that full information on the approval of the study protocol must also be provided in the manuscript.

Plants

Seed stocks	*Report on the source of all seed stocks or other plant material used. If applicable, state the seed stock centre and catalogue number. If plant specimens were collected from the field, describe the collection location, date and sampling procedures.*
Novel plant genotypes	*Describe the methods by which all novel plant genotypes were produced. This includes those generated by transgenic approaches, gene editing, chemical/radiation-based mutagenesis and hybridization. For transgenic lines, describe the transformation method, the number of independent lines analyzed and the generation upon which experiments were performed. For gene-edited lines, describe the editor used, the endogenous sequence targeted for editing, the targeting guide RNA sequence (if applicable) and how the editor was applied.*
Authentication	*Describe any authentication procedures for each seed stock used or novel genotype generated. Describe any experiments used to assess the effect of a mutation and, where applicable, how potential secondary effects (e.g. second site T-DNA insertions, mosiacism, off-target gene editing) were examined.*
